# MicroRNA-1 attenuates the growth and metastasis of small cell lung cancer through CXCR4/FOXM1/RRM2 axis

**DOI:** 10.1186/s12943-022-01695-6

**Published:** 2023-01-04

**Authors:** Parvez Khan, Jawed Akhtar Siddiqui, Prakash G. Kshirsagar, Ramakanth Chirravuri Venkata, Shailendra Kumar Maurya, Tamara Mirzapoiazova, Naveenkumar Perumal, Sanjib Chaudhary, Ranjana Kumari Kanchan, Mahek Fatima, Md Arafat Khan, Asad Ur Rehman, Imayavaramban Lakshmanan, Sidharth Mahapatra, Geoffrey A. Talmon, Prakash Kulkarni, Apar K. Ganti, Maneesh Jain, Ravi Salgia, Surinder Kumar Batra, Mohd Wasim Nasser

**Affiliations:** 1 Department of Biochemistry and Molecular Biology, University of Nebraska Medical Center, Omaha, NE 68198 USA; 2 Department of Medical Oncology and Therapeutics Research, City of Hope National Medical Center and Beckman Research Institute, Duarte, CA 91010 USA; 3 Fred and Pamela Buffett Cancer Center, University of Nebraska Medical Center, Omaha, NE 68198 USA; 4 Department of Pediatrics, University of Nebraska Medical Center, Omaha, NE 68198 USA; 5 Department of Pathology and Microbiology, University of Nebraska Medical Center, Omaha, NE 68198 USA; 6 Division of Oncology-Hematology, Department of Internal Medicine, VA-Nebraska Western Iowa Health Care System, University of Nebraska Medical Center, Omaha, NE 68198 USA; 7 Eppley Institute for Research in Cancer and Allied Diseases, University of Nebraska Medical Center, Omaha, NE 68198 USA

**Keywords:** Small cell lung cancer, microRNAs, CXCR4, FOXM1, RRM2, Neuroendocrine carcinoma

## Abstract

**Background:**

Small cell lung cancer (SCLC) is an aggressive lung cancer subtype that is associated with high recurrence and poor prognosis. Due to lack of potential drug targets, SCLC patients have few therapeutic options. MicroRNAs (miRNAs) provide an interesting repertoire of therapeutic molecules; however, the identification of miRNAs regulating SCLC growth and metastasis and their precise regulatory mechanisms are not well understood.

**Methods:**

To identify novel miRNAs regulating SCLC, we performed miRNA-sequencing from donor/patient serum samples and analyzed the bulk RNA-sequencing data from the tumors of SCLC patients. Further, we developed a nanotechnology-based, highly sensitive method to detect microRNA-1 (miR-1, identified miRNA) in patient serum samples and SCLC cell lines. To assess the therapeutic potential of miR-1, we developed various in vitro models, including miR-1 sponge (miR-1Zip) and DOX-On-miR-1 (Tet-ON) inducible stable overexpression systems. Mouse models derived from intracardiac injection of SCLC cells (miR-1Zip and DOX-On-miR-1) were established to delineate the role of miR-1 in SCLC metastasis. In situ hybridization and immunohistochemistry were used to analyze the expression of miR-1 and target proteins (mouse and human tumor specimens), respectively. Dual-luciferase assay was used to validate the target of miR-1, and chromatin immunoprecipitation assay was used to investigate the protein-gene interactions.

**Results:**

A consistent downregulation of miR-1 was observed in tumor tissues and serum samples of SCLC patients compared to their matched normal controls, and these results were recapitulated in SCLC cell lines. Gain of function studies of miR-1 in SCLC cell lines showed decreased cell growth and oncogenic signaling, whereas loss of function studies of miR-1 rescued this effect. Intracardiac injection of gain of function of miR-1 SCLC cell lines in the mouse models showed a decrease in distant organ metastasis, whereas loss of function of miR-1 potentiated growth and metastasis. Mechanistic studies revealed that CXCR4 is a direct target of miR-1 in SCLC. Using unbiased transcriptomic analysis, we identified CXCR4/FOXM1/RRM2 as a unique axis that regulates SCLC growth and metastasis. Our results further showed that FOXM1 directly binds to the RRM2 promoter and regulates its activity in SCLC.

**Conclusions:**

Our findings revealed that miR-1 is a critical regulator for decreasing SCLC growth and metastasis. It targets the CXCR4/FOXM1/RRM2 axis and has a high potential for the development of novel SCLC therapies.

**Graphical Abstract:**

MicroRNA-1 (miR-1) downregulation in the tumor tissues and serum samples of SCLC patients is an important hallmark of tumor growth and metastasis. The introduction of miR-1 in SCLC cell lines decreases cell growth and metastasis. Mechanistically, miR-1 directly targets CXCR4, which further prevents FOXM1 binding to the *RRM2* promoter and decreases SCLC growth and metastasis.

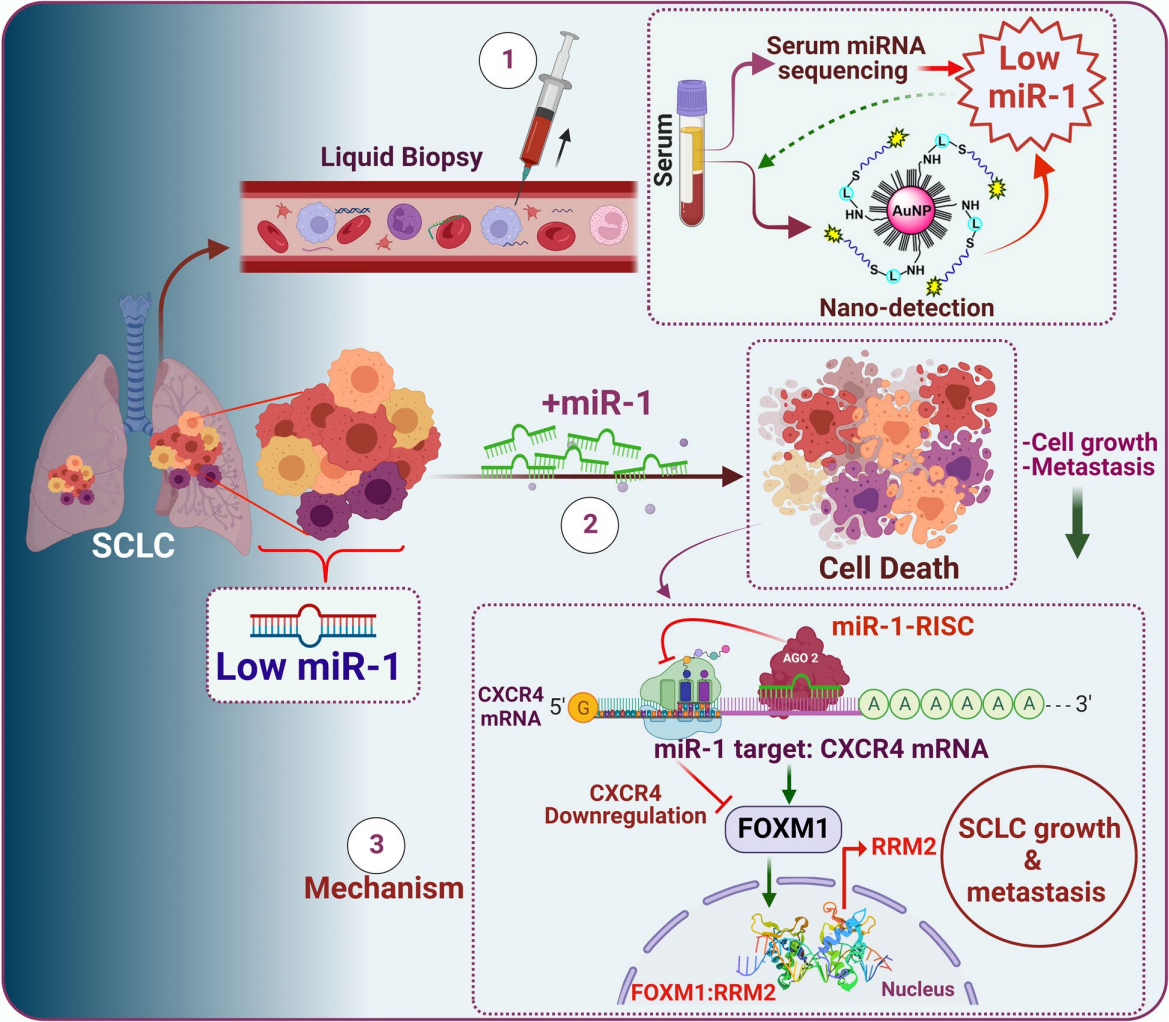

**Supplementary Information:**

The online version contains supplementary material available at 10.1186/s12943-022-01695-6.

## Background

Small cell lung cancer (SCLC) is a metastatic neuroendocrine disease that remains among the most lethal type of solid tumors [1]. Initially, SCLC respond to conventional chemotherapy (cisplatin/carboplatin/etoposide), with a response rate of ~ 60–70%; however, a majority of the SCLC patients develop resistance to front-line therapies, experience relapse, and die in a matter of months [2, 3]. Due to high metastasis, late diagnosis, and recalcitrant behavior of SCLC, a limited number of therapies are available and despite the incorporation of immunotherapy to platinum-based front-line therapies, improvements in overall survival or outcomes of SCLC remain poor [4–6].

Identifying potential therapeutic targets in SCLC remains challenging. A major limiting factor is the high mutational load leading to the inactivation or deletion of two major tumor suppressor genes (*RB1* and *TP53*) [7, 8]. Along with the dysregulation of protein-coding genes, non-coding genes were also found dysregulated in various cancer types, including SCLC [9]. Only 2% of transcribed genes encode functional proteins and untranslated genes constitute a major part of the human genome with important gene regulatory roles [9]. One such important class of genes encode microRNAs (miRNAs); these are the small nucleotide sequences with a key regulatory impact on gene expression [10, 11]. The miRNAs bind to complementary sequences on the target mRNA to preclude translation through a sequence-specific RNA-interference (RNAi) mechanism [11]. Simultaneously, a single miRNA can have multiple genomic targets and multiple miRNAs regulate one or more hallmarks of cancer; thus, microRNAs have the potential to regulate different molecular pathways [12, 13]. More than 2800 mature human miRNA sequences are currently annotated in public databases like miRBase V22, and their sequences are evolutionarily conserved [14].

Interestingly, miRNAs can behave as tumor suppressors and tumor promoters [15, 16]. Several studies have demonstrated that miRNAs regulate cell viability, growth, migration, angiogenesis, and apoptosis [10]. A single miRNA can regulate the expression of different genes at transcriptional or translational levels; this provides a valuable platform to investigate the role of miRNAs and their targets in the pathogenesis of various cancers, including SCLC. In the present study, employing miRNA sequencing and various screening analyses, we identified and rationalized the investigation of tumor suppressor microRNA-1 (miR-1) in SCLC. Previously, miR-1 has been reported to play a major role in cardiomyocyte differentiation and smooth muscle development [17, 18]. *MiR-1* is highly conserved throughout the mammalian system and acts as a consistent tumor suppressor gene [17, 19]. However, the impact of miR-1 on SCLC pathogenesis remains unexplored. Here, we profiled miR-1 expression and functional outcomes in SCLC and showed that miR-1 downregulation is associated with SCLC growth and metastasis. Further, we investigated CXCR4/FOXM1/RRM2 axis as a novel target of miR-1 both in vitro and in vivo. Its overexpression or targeted delivery can be a potential therapeutic strategy to improve the outcomes of SCLC.

## Material and methods

### Cell lines and maintenance

The human SCLC cell line SBC3 was gifted by Dr. Takashi Kijima (Osaka University, Japan), and SBC5 was obtained from the Japanese Collection of Research Bioresources (JCRB) Cell Bank, National Institutes of Biomedical Innovation, Health and Nutrition, Osaka, Japan. BEAS-2B, DMS273, DMS53, NCI-H82, NCI-H526, NCI-H69 and Colo668 were originally obtained from ATCC (Rockville, MD, USA). All the cell lines were routinely maintained and cultured in standard culture conditions, in a CO_2_ incubator at 37 °C, and regularly validated using STR profiling and checked for mycoplasma infections prior to experiments. For routine maintenance, SBC3, SBC5, NCI-H82, NCI-H69, NCI-H526, DMS273, DMS53, and Colo668 cells were cultured in RPMI-1640 medium supplemented with 1500 mg/L sodium bicarbonate, 10% fetal bovine serum (Sigma Cat#12303C), 1% penicillin/streptomycin cocktail solution (Invitrogen Cat#15,140–122), 1% sodium pyruvate (Invitrogen Cat#11,360,070), and 1% L-glutamine (Invitrogen Cat#25,030–081).

### DNA-AuNPs nanoprobe for the detection of miR-1 in serum and cell lines

Except for the cell-isolated total RNA sample, the in situ (RNA isolation-free) detection of the serum miRNA was performed after treating the serum with the denaturation buffer (1X TAE buffer, 2.0 M NaCl, 10% IGEPAL CA-630, 0.01U/µL RNase inhibitor, 40 µg of proteinase K (from a 20 mg/mL stock). The denaturation step ensured that serum miRNAs were freed from transport vesicles/exosomes and stabilizing AGO2 protein complexes, and it denatured the background proteins [20, 21]. Briefly, the serum samples were incubated for 30 min in an equal volume of denaturation buffer and heated to 95 °C for 5 min. The protein pellet was removed by quick centrifugation (6000 rpm, 6 min, room temperature). Samples representing a starting serum volume of 20 µl were reacted with 2.1 pM DNA-AuNPs [DNA-probe = 2.49 nM), DSN enzyme (0.03 U/µL, Cat#70600-202 from Arcticzyme Technologies) in DSN buffer (50 mM Tris–HCl, 5 mM MgCl_2_, 10 mM DTT, RNase inhibitor 0.01U/µL pH 8.0) in a 50 µL volume (384-well) overlaid with silicone oil (6μL). In the same plate, we also quantified the known concentrations of the synthetic miR-1 oligos (2.5-1000 pM) to construct a standard curve representing miR-1 concentration using the optimized conditions and instrumental settings as mentioned in Table S1-S4. Finally, to estimate the miR-1 concentration, the enhanced fluorescence intensity slope (before reaching signal plateau) was determined and compared with the standard curve (fluorescence ratio (F/F_0_-1) vs. [miR-1]).

### In-situ hybridization

The expression level of miR-1 was determined by employing in situ hybridization (ISH). For this, we used a commercially available, single-color human or mouse-specific 20-paired double-Z oligonucleotide miR-1 probe (Advanced Cell Diagnostics) as described previously [22]. The images were captured using a 40 × and 100 × Bright-field objective.

### Lentivirus production

Lentivirus particles were generated using the Lipofectamine-2000 co-transfection method in HEK293T cells. Briefly, the respective lentivirus-based expression vectors of miR-1, miR-1Zip, and control vector were co-transfected with packaging vectors from Addgene; pMDLg/pRRE #12,251, pRSV-Rev #12,253, and envelope expressing plasmid pMD2.G #12,259. Culture supernatant containing lentiviral particles was collected after 48 h and 72 h of transfection, centrifuged to clear the cell debris, filtered through 0.45-micron sterile filters, aliquoted, and stored at -80°C until used.

### Generation of miR-1 inducible and miR-1Zip system

Stable, inducible expression clones of miR-1 were generated in SBC5 and NCI-H69 cell line using 3^rd^ generation lentiviral system. We performed a dual transduction method using rtTA along with pLV[Tet]mCherry:T2A:Puro > TRE3G > FLAG/hmiR-1/HA:T2A:Luciferase or pLV[Tet]-mCherry:T2A:Puro > TRE3G- > Luciferase vectors, procured from VectorBuilder, TX. The expression vector consecutively expresses mCherry, whereas the luciferase expression is under the control of Tet-inducible promoter and used for in vivo imaging. Nearly 1 × 10^5^ SBC5 or NCI-H69 cells were seeded in a six-well plate and transduced with lentiviral particles (with 8 μg/ml polybrene) for transduction. After 24–48 h incubation, the transduced cells were replenished with the fresh cell culture medium. The transduction efficiency was monitored by visualizing the cells for mCherry expression using fluorescence microscopy. Finally, the mCherry positive cells were sorted on FACS Aria II (B.D. Biosciences), and consequently transduced with rtTA-lentiviral particles (for the doxycycline-induced activation of TRE3G promoter by the transactivator Tet3G). The anti-miR-1 microRNA (miR-1Zip) lentivirus expression vector was obtained from System Biosciences, California, USA (miRZip-1 anti-miR-1 microRNA construct, Catalogue # MZIP1-PA-1, having T2A: Puro > CMV:copGFP > H1:miRZip-1 anti-miR-1), which was transduced in SBC3 cell lines, as described above.

### Small RNA library preparation and miRNA sequencing from serum samples of SCLC patients

The cell-free total RNA, including miRNAs was isolated using miRNeasy serum/plasm kit (Qiagen, Germantown, MD, Cat # 217,184) by following the manufacturer’s protocol. All libraries were prepared using the Illumina TruSeq Small RNA protocol with minor modifications following the manufacturer’s instructions with 12 cycles of PCR amplification after ligation of the 3′ and 5′ adapters. This protocol is ideal for the investigation of small RNA species, as it takes advantage of the structure of most small RNA molecules by ligating specific adapters to the 5′-phosphate and 3′-hydroxyl group, which are molecular signatures of their biogenesis pathway. Individual libraries were prepared using a unique index primer to allow for the pooling of multiple samples prior to sequencing. The library was quantified using qPCR. Sequencing was performed on a Hiseq2500 (Illumina Inc., San Diego, CA), and image processing and base calling were conducted using Illumina's pipeline.

### RNA-sequencing and deferential gene expression analysis

SBC3/SBC3-miR-1Zip, SBC5/SBC5-miR-1-Tet-ON (-DOX/ + DOX) cells were lysed using RNA lysis buffer provided with the mirVana RNA isolation kit (Cat#AM1561, Thermo Fischer Scientific). Total RNA was isolated by following the manufacturer’s protocol. Whole transcriptome analysis (RNA sequencing) was performed on SBC3/SBC3-miR-1Zip, SBC5/SBC5-miR-1-Tet-ON (-DOX/ + DOX) cells at Sequencing Core Facility, City of Hope Comprehensive Cancer Center and Beckman Research Institute. RNA quality was determined using Agilent Bioanalyzer (Agilent Technologies, Inc.) and RNA samples with integrity numbers (RINs) of 10 were used for further analyses. Library preparation, PCR amplification, size distribution, library quantification, and sample loading were performed as described previously [23]. Sequencing was performed on a HiSeq2500 sequencer (Illumina Inc., San Diego, CA) in rapid mode. A single read, 50 cycle, sequencing run and onboard clustering and V2 chemistry were used. Raw files for RNA-seq were submitted NCBI SRA (accession number PRJNA900568).

### Chromatin immunoprecipitation and qPCR

Chromatin immunoprecipitation (ChIP) was performed with minor modifications [24]. Briefly, 15 × 10^6^ cells were cross-linked with 1% formaldehyde for 10 min at room temperature. The cross-linking was stopped by adding 0.125 M glycine for 5 min, and cells were washed with chilled PBS (2X), scraped, and collected by centrifugation (820 × g, 5 min, 4 °C). The enriched ChIP DNA was analyzed by quantitative real-time PCR using Syber green on the CFX Connect Real-Time PCR Detection System (Bio-Rad Laboratories, Hercules, CA). The primers used in the study are-P1 F/R, P2 F/R, and P3 F/R *(details provided in* supplementary information*)*. The coimmunoprecipitated gene fragments were represented as a fold enrichment method based on Ct values (2^−(Ct(FOXM1 IP) – Ct(IgG))^).

### CXCR4 3'-UTR dual luciferase assay

CXCR4 3'-UTR luciferase assay was performed as described previously [25]. Following 48 h of CXCR4 3'-UTR-wild-pGL3/3'-UTR-mutant-pGL3 transfection, luciferase activity was measured using the Dual-Luciferase Reporter Assay System (Promega, Madison, WI) using a Luminometer (Agilent, Biotek, CA, USA).

### SCLC cell line xenografts studies

SBC3 and SBC3-miR-1Zip cells were transduced with firefly luciferase-expressing construct and resuspended at 3 × 10^7^ cells/ml in PBS. Nearly 3 × 10^6^ cells (SBC3 or SBC3-miR-1Zip) were implanted subcutaneously into bilateral flanks of 8-week-old NSG mice (both male and female). The mice were followed and monitored daily, and tumor growth was followed by an IVIS imaging system and caliper-based measurements. The tumor volume measurements were carried out using the relation: tumor volume (mm^3^) = (tumor width)^2^ × length/2. All mouse experiments/procedures were duly approved and performed as per Institutional animal care and use committee (IACUC) guidelines in accordance with the National Institute of Health (NIH). The animals were maintained as per IACUC guidelines in the University of Nebraska Medical Center (UNMC) animal house facility.

### Intracardiac injections

#### SBC3 and SBC3-miR-1Zip cells

To determine the impact of miR-1 inhibition on the metastatic potential of SCLC cells, intracardiac injections of SBC3 and SBC3-miR-1Zip cells were performed as described previously [22]. Briefly, 1 × 10^5^ SBC3-luciferase or SBC3-miR-1Zip-luciferase cells were injected into the left ventricle of female or male NSG mice. The mice were monitored weekly for metastases through luciferase-luciferin bioluminescence measurement using an IVIS imaging system. Before imaging, luciferin (150 mg/kg in sterile PBS) was injected intraperitoneally. Reaching the experiment endpoint (~ 6 weeks), mice were euthanized, and all major organs (lung, liver, spleen, brain, pancreas, intestine, adrenal, ovary, hindlimb bones) were harvested within 10 min of luciferin injection, and *ex-vivo* IVIS imaging was performed to see metastasis through luciferin-luciferase bioluminescent imaging. Serum was obtained from the collected blood after a cardiac puncture at the time of sacrifice for analyzing miR-1 expression.

#### miR-1 inducible SBC5 cells

To study the impact of miR-1 overexpression on SCLC growth and metastasis, intracardiac injections of Tet inducible miR-1 overexpressing SBC5 cells were performed. Briefly, 1 × 10^5^ SBC5 (-DOX-off)/SBC5-DOX-On-miR-1 cells were injected into the left ventricle of NSG mice (both male and female). After two weeks of intracardiac injections, mice were randomized into two groups, -DOX (without doxycycline) and + DOX (with doxycycline). In the case of + DOX animal group, mice were kept on special DOX feed (ENVIGO, Cat#TD.08541, Rodent Diet 2018, 625 Dox, R) or water (doxycycline, 3.2 g/liter, and sucrose 30 g/liter) to induce miR-1 overexpression whereas -DOX mice were on a respective control diet (ENVIGO, Cat#TD.00588, Global 18% Protein Rodent Diet-Control). The doxycycline-sucrose solution was changed every 2 to 3 days throughout the experiment. The metastases were monitored through luciferin bioluminescence measurement of luciferase-expressing cells using the IVIS system. Luciferin (150 mg/kg in sterile PBS) was injected intraperitoneally before imaging. Serum was obtained from the collected blood after a cardiac puncture at the time of sacrifice for analyzing miR-1 expression.

### Survival studies

A total of 20 mice (both male and female) intracardially injected with SBC5 (-DOX-off)/SBC5-DOX-On-miR-1 were randomized and switched to DOX diet (with miR-1) or -DOX (without miR-1) groups, and the survival of mice was monitored for nearly six months. Kaplan–Meier survival estimate was used to determine the median overall survival, and the statistical significance was ranked by *P*-value < 0.05.

### Statistical analysis

All graphs were presented as mean ± SEM unless otherwise indicated. The statistical tests to perceive the differences between the two groups were implemented using a two-tailed unpaired Student *t*-test or a two-tailed Mann–Whitney test (as specified in the respective figure legends) or ordinary one-way ANOVA was used to consider the significance in more than two groups, (specified in the respective figure legends). The Kaplan–Meier method and Log-rank test were used for the miR-1 mediated mouse survival analyses. *P* values were considered significant if less than 0.05. The asterisks used to specify significance correspond with *, *P* < 0.05; **, *P* < 0.01; *** *P* < 0.001; ****, *P* < 0.0001.

## Results

### Overall microRNA expression landscape in SCLC identifies miR-1 as a tumor suppressor gene in SCLC

**Fig. 1:**
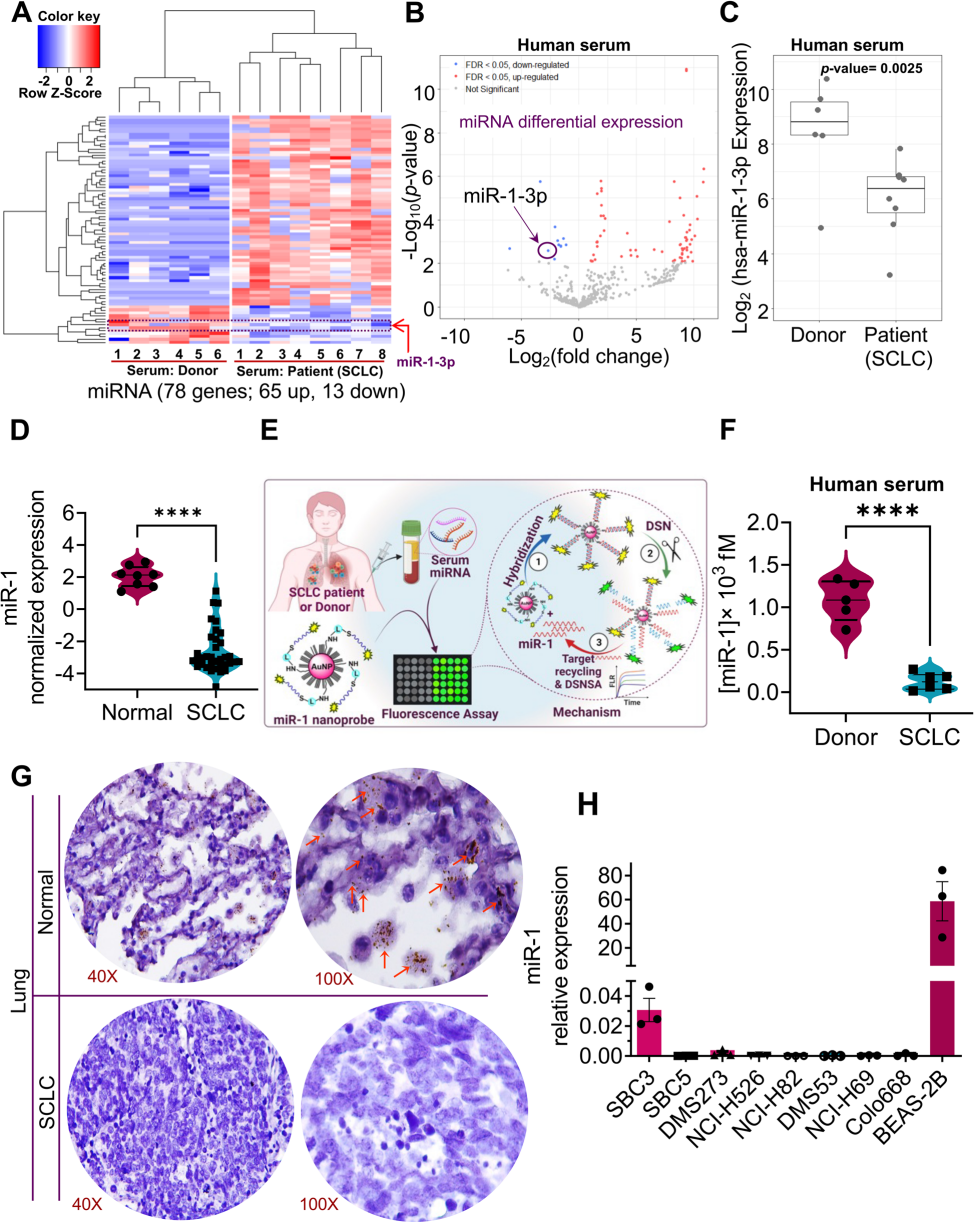
MicroRNA profiling in SCLC patient serum identifies miR-1-3p (miR-1) is downregulated in SCLC and validated by DNA-AuNP˗based in situ detection and quantification of serum miR-1. **A** The clustered heatmap of top differentially expressed genes for microRNAs in the serum samples of 6 healthy donors and 8 SCLC patient samples. The red and blue strips indicate upregulated and downregulated miRNAs. **B** The volcano plot showed that miR-1 was one of the downregulated miRNAs. **C** Comparison of log_2_ fold change expression of miR-1 in the serum samples of 6 healthy donors and 8 SCLC patient samples. **D** Violin plots comparing the expression profile of miR-1 between normal lung tissues (*n* = 8) and SCLC tumor tissues (*n* = 35) in the publicly available gene expression data of human cancers (GSE19945). **E** Schematic of Au-nanoprobe (DNA-AuNPs) mediated detection of serum miR-1 in the denatured human serum samples. **F** Violin with point graph representing the quantitative expression of serum miR-1 in the donor (normal control) serum vs SCLC patient serum. *p*-values are the results of Welch’s t-test. **G** Representative in situ hybridization of miR-1 on SCLC tissue microarray (BS04116a, US Biomax, Inc., representing the pathology grade tumor tissues of 55 cases/100 cores for SCLC and ten cores for normal lung tissues), stained with RNAscope miR-1 detection probe. **H** Expression of miR-1 in a panel of human SCLC cell lines analyzed by Taq-man qPCR assay

MicroRNAs or non-coding RNAs are important molecules that regulate the expression pattern of multiple genes, including tumor suppressors and tumor promoters [26, 27]. Therefore, apart from identifying protein targets, microRNAs also provide a means to identify molecules modulating the various aspects of cancer and puts forward the potential of identifying tumor suppressor microRNAs as a useful strategy to develop or identify novel therapeutic agents. To understand the expression profile of microRNAs in SCLC, we performed miRNA sequencing from serum samples of eight SCLC patients and six healthy donors. The advantage of using serum samples is the secretory nature of miRNAs, which exist in exosomes and argonaute (AGO) proteins in circulation/serum that can be easily detected using noninvasive techniques [21, 28, 29]. We observed a differential expression pattern of miRs (65 up/13 down) in SCLC/donor serum samples, and interestingly, we observed a significant downregulation of miR-1 in SCLC patients compared to healthy donors (Fig. 1A-C, supplementary Fig. S1A-C). To further understand miR-1 expression in SCLC and to validate the observation of miRNA-Seq, we also performed bulk RNA-Seq analysis in the tumor tissues of SCLC patients and normal lung tissues (GSE19945). We observed substantial downregulation of miR-1 in the SCLC tumors compared to normal lung tissues (Fig. 1D, supplementary Fig. S2).

Further, to analyze miR-1 expression in SCLC, we developed an advanced and highly sensitive nanoprobe-based biosensing assay that performs the absolute quantification of miR-1 in serum or cell line samples (Fig. 1E). For this, we synthesized and characterized a DNA-probe-functionalized Au-nanoprobe (DNA-AuNPs) that can be used to detect miR-1 from cell-derived total RNA or directly from serum or plasma. The synthesis and characterization are provided in supplementary information and supplementary figures S3-S4. Given that most miRNAs exist with AGO2 protein forming a RNA-induced silencing complex (RISC) and exosomes [21, 29, 30], we performed a gold nanoprobe-mediated in situ (RNA isolation free) detection of miR-1 in the serum samples of human SCLC patients and healthy donors (Fig. 1F, supplementary Fig. S3-S4). Notably, to enhance the accuracy of serum miRNAs assay, a protein denaturation step was performed using short treatment of the denaturation buffer on the serum followed by heat inactivation and removal of excess proteins (for details, see supplementary information) to facilitate the availability of miRNAs for biosensing purpose. Interestingly, we observed that miR-1 expression was drastically decreased in the serum samples of SCLC patients compared to healthy donors (Fig. 1 F).

In addition, to determine the clinical significance of miR-1 expression, we performed *in-situ* hybridization on SCLC tissue microarray (Cat#, BS04116a, US Biomax, Inc. Derwood, MD), representing the pathology grade tumor tissues of 55 cases/100 cores for SCLC and ten cores for normal lung tissues. Using a miR-1-specific probe, the *in-situ* hybridization results validated the observations of miRNA-Seq and miR-1 serum profiling data that showed decreased expression of miR-1 in SCLC tumor tissues compared to normal lung tissues (Fig. 1G). Further, to determine miR-1 expression in SCLC cell lines, we performed a Taq-man-based miR-1 expression assay using a panel of SCLC cell lines (SBC3, SBC5, DMS273, NCI-H526, NCI-H82, DMS53, NCI-H69, and Colo668) and non-tumorigenic lung epithelial cells (BEAS-2B). Consistent with the bulk gene expression data, serum miRNAs sequencing, and nanoprobe-mediated expression profile of serum miR-1, decreased to null expression of miR-1 was observed in SCLC cell lines compared to non-tumorigenic lung epithelial cells (Fig. 1H). Together, our results suggest that downregulation of miR-1 accompanying SCLC, and consistent with the previous studies in other cancers [19, 31], establishes miR-1 as a tumor suppressor in SCLC.

### miR-1 overexpression decreases the growth and metastasis of SCLC cells, whereas miR-1 inhibition potentiates cell growth and migration of SCLC cells

To investigate the role of miR-1 in the growth and metastasis of SCLC cells, we infected miR-1 expressing cell line (SBC3) with lentivirus containing miR-1Zip-GFP that was used to develop stable knockdown in the SBC3 cell line. For making a stable miR-1 overexpression model, we infected two miR-1 null SCLC cell lines (SBC5 and NCI-H69) with lentivirus that constitutively expresses mCherry, whereas the expression of miR-1 and luciferase was under the control of doxycycline (DOX) inducible cassette. We selected the positive clones using puromycin selection and single-cell sorting using GFP (for miR-1Zip) or mCherry (for DOX-On-miR-1) as markers. The DOX-On-miR-1 cells were further infected with lentivirus expressing rtTA having hygromycin as a selectable marker. In all the experiments, lentiviral-infected cells were maintained under the selection of the drug (puromycin/hygromycin) suitable for the corresponding lentiviral vector.

**Fig. 2:**
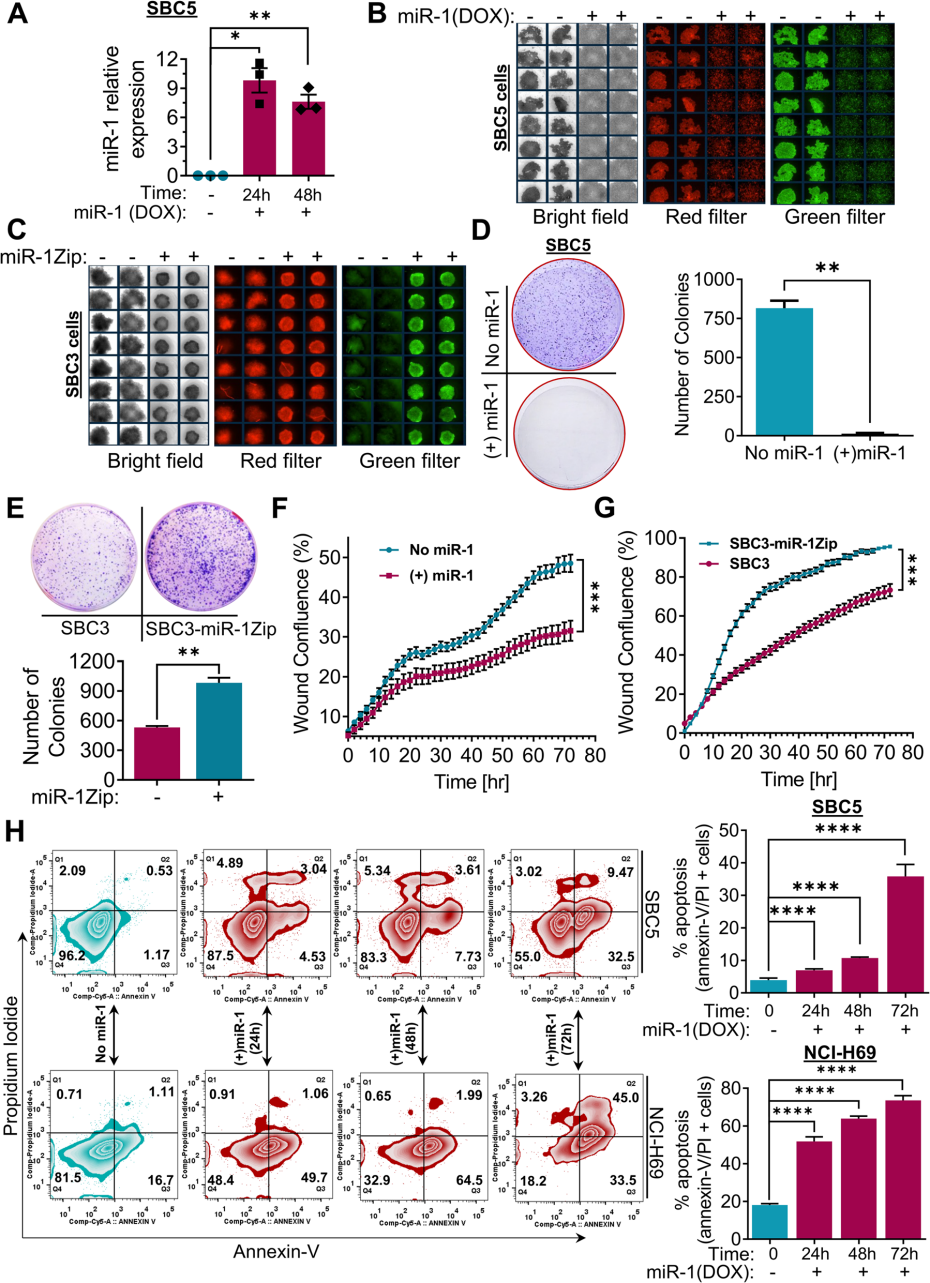
miR-1 modulates growth and migration of SCLC cells. **A** miR-1 expression in DOX-On-miR-1 SBC5 cells induced with DOX for 24–48 h assessed by TaqMan-based qPCR assay. RNU6B was used to normalize the gene expression. Representative images from sphere formation assay from, (**B**) SBC5 no miR-1(-DOX-Off) and + miR-1 (+ DOX-On), (**C**) SBC3 and SBC3-miR-1Zip cells. Representative images for colony formation assay from, (**D**) SBC5 no miR-1(-DOX-Off) and + miR-1 (+ DOX-On) cells, the right panel showed the quantification of number of colonies from *n* = 3 biological replicates, (**E**) SBC3 and SBC3-miR-1Zip cells, the lower panel showed the quantification of the number of colonies from *n* = 3 biological replicates. Quantification of percent wound closure area from real-time monitoring for cell migration assay of, (**F**) SBC5 no miR-1(-DOX-Off) and + miR-1 (+ DOX-On) cells, (**G**) SBC3 and SBC3-miR-1Zip cells. **H** FACS analysis of the apoptotic potential of miR-1 in SBC5/NCI-H69 cells under no miR-1(-DOX-Off) and + miR-1 (+ DOX-On) conditions as monitored for 24–72 h. The cells were induced for miR-1 expression for the indicated period (24-72 h) and stained with annexin-V/PI to analyze apoptosis induction by flow cytometry. Quantification of apoptosis was presented in the right panels (from *n* = 3 biological replicates). Statistical significance was considered using a two-tailed Student’s t-test, except in **H**, where ordinary one-way ANOVA was used. *, *p* < 0.05; **, *p* < 0.01; ***, *p* < 0.001; ****, *p* < 0.0001

Before starting the functional studies, we first determined the miR-1 expression using miR-1 specific Taq-man qRT assay in SBC5-DOX-On miR-1 cells and observed that these cells showed significant upregulation in miR-1 expression following the 24–48 h induction with DOX compared to non-induced (-DOX-off) SBC5 cells (Fig. 2A). To monitor the effect of miR-1 on SCLC cells, SBC5-DOX-On miR-1 cells were plated in a low attachment cell culture plate and allowed to form spheres. We monitored and analyzed the growth of spheres formed by SBC5 (-DOX-off) vs. SBC5-DOX-On miR-1 cells, and as expected [31, 32], once we induced miR-1 expression in SBC5 cells using DOX-On miR-1, it suppressed the sphere formation ability of SBC5 cells (Fig. 2B). In a complementary set of experiments on SBC3 and SBC3-miR-1Zip cells, it was found that miR-1 sponging (via miR-1Zip) supported the sphere-forming ability of SBC3 cells compared to parental SBC3 cells (Fig. 2C). In another variation of cell growth experiments, we also examined the role of miR-1 in colony formation by SCLC cells and found that miR-1 overexpression significantly decreased the colony formation in SBC5 cells, whereas SBC3-miR-1Zip cells had a higher colony-forming ability compared to parental cells (Fig. 2D-E).

Next, to see the impact of miR-1 on the migration properties of SCLC cells, we employed a live cell monitoring approach using IncuCyte coupled live-cell imaging. We seeded the SBC5(-DOX-off)/SBC5-DOX-On-miR-1 and SBC3/SBC3-miR-1Zip cells in a 96-well plate and simultaneously created a homogenous wound using the wound creating apparatus provided with the IncuCyte machine. We followed wound closure ability of SBC5(-DOX-off)/SBC5-DOX-On-miR-1 (without or with miR-1) and SBC3/SBC3-miR-1Zip cells in real-time for 72 h and found that miR-1 decreased the wound closure ability of SBC5 cells, and miR-1 sponging enhanced the wound closure or migratory properties of SBC3 cells as revealed by live-cell monitoring, scratch wound assay, and transwell migration studies (Fig. 2F-G & supplementary Fig. S5 A-D). We were then interested in discerning the apoptotic potential of miR-1 in SCLC cells (SBC5 and NCI-H69). For this, the DOX-On-miR-1 SBC5 and NCI-H69 cells were plated, and miR-1 expression was induced (+ DOX) for the different time periods (24–72 h), and apoptosis quantification was performed through flow cytometry-based annexin-V/PI staining. We observed that miR-1 overexpression induced apoptosis-like morphological changes in the SCLC cells (supplementary Fig. S6A-B). Further, we found a time-dependent significant increase in the percent apoptosis of miR-1 induced SBC5 and NCI-H69 cells compared to their respective -DOX-off controls (Fig. 2H). Thus, overall results showed that miR-1 decreased the sphere formation and cell migration properties of SCLC cells and induced apoptosis; on the other hand, miR-1 sponging enhanced sphere-forming and cell migration abilities of SCLC cells.

### miR-1 modulates tumor growth and metastasis in SCLC xenografts

**Fig. 3:**
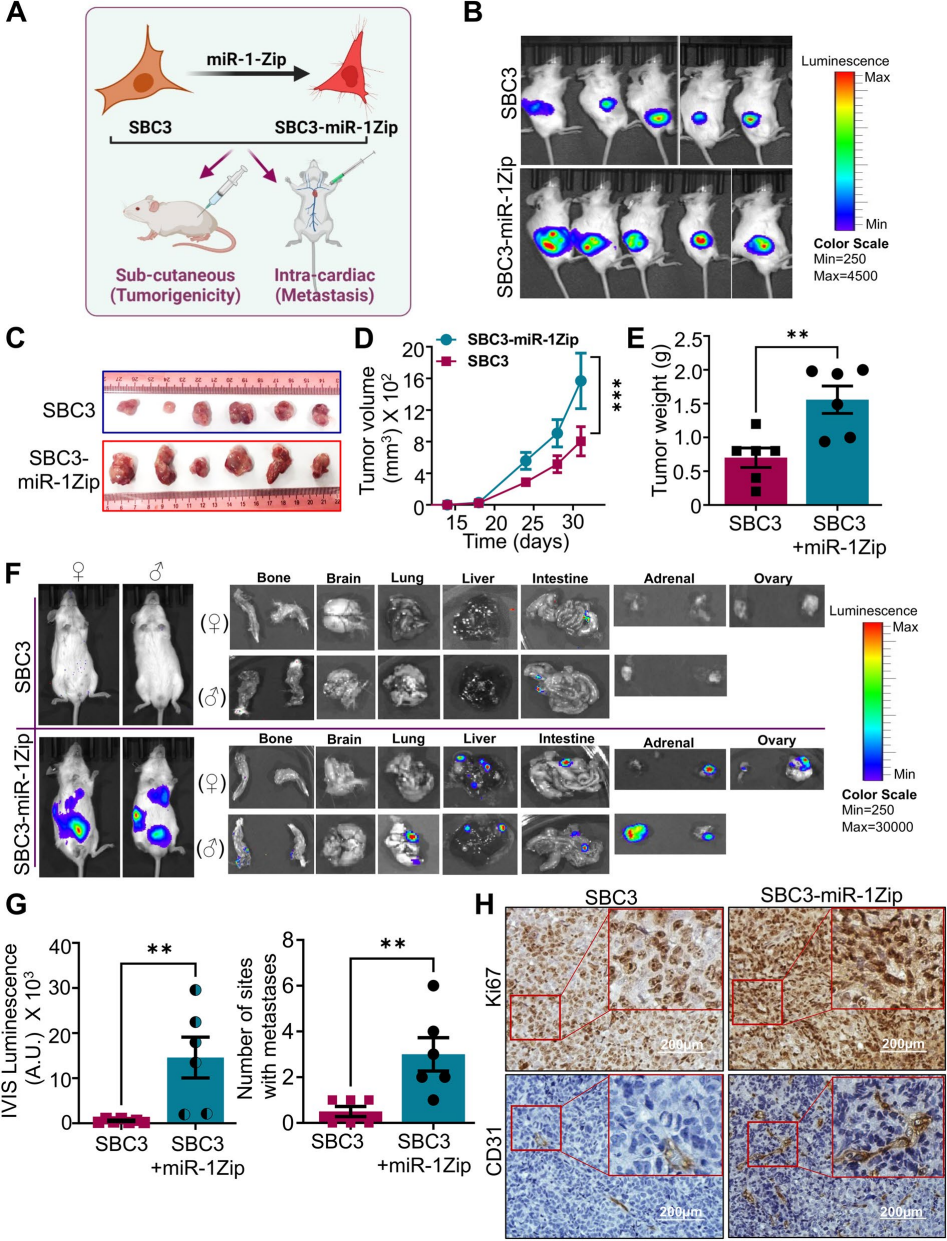
Downregulation of miR-1 or sponging drives a highly aggressive phenotype in SCLC cells. **A** Schematic of SBC3 and SBC3-miR-1Zip subcutaneous and intracardiac xenografts in NSG mice for tumorigenesis and metastasis studies. **B** Representative IVIS image of NSG mice injected with subcutaneous xenografts of SBC3 and SBC3-miR-1Zip cells expressing luciferase. **C ***Ex-vivo* images of tumors from SBC3 and SBC3-miR-1Zip xenografts. **D** Quantification of time-dependent tumor growth (SBC3 and SBC3-miR-1Zip tumors) in volume as obtained from caliper measurements. **E** Quantification of tumor growth in terms of weight (g) at the end of experiments. **F** Representative IVIS images of mice bearing intracardiac xenografts on the end day of the experiment just before the time of sacrifice (one female and one male mice), with *ex-vivo* IVIS imaging of major organs for the metastasis of SBC3 and SBC3-miR-1Zip cells such as lung, liver, brain, bone, stomach, intestine, pancreas, spleen, adrenal and ovary. Mice were injected with luciferin i.p. (150 mg/kg in sterile PBS) 5 min before euthanasia; following that the major organs were collected and scanned for metastatic lesions using IVIS imaging. **G** Quantification of the IVIS luminescence and number of metastases sites from each mouse intracardially injected with SBC3 and SBC3-miR-1Zip group. **H** Representative IHC of SBC3 and SBC3-miR-1Zip tumor sections for Ki67 and CD31, highlighted with red box provided in higher-magnification or zoom inset. Scale bars represent 200 µm (with successive magnification)

To probe deeper into the role of miR-1 in SCLC growth and metastasis, we performed tumorigenesis and metastasis studies with luciferase labeled SBC3 and SBC3-miR-1Zip cells using subcutaneous and intracardiac xenograft models in immunodeficient NOD/SCID gamma (NSG) mice (Fig. 3A). For tumorigenesis, the SBC3-luciferase and SBC3-miR-1Zip-luciferase cells were subcutaneously injected into the right flank of NSG mice (*n* = 6 per group with equal number of male and female mice) and monitored for tumor growth using IVIS-imaging (Fig. 3B). We found that the growth of SBC3-miR-1Zip xenografts was significantly higher compared to SBC3-parental cells suggesting that miR-1 sponging enhanced the tumorigenesis of SBC3 cells (Fig. 3C-E).

To determine the effect of miR-1 sponging on the metastatic potential of SCLC cells, we injected SBC3-luciferase and SBC3-miR-1Zip-luciferase cells intracardially in NSG mice, and we observed higher metastases in animals injected with SBC3-miR-1Zip cells (Fig. 3F). Remarkably, significant metastasis was observed in the liver, lung, intestine, ovary (female mice), and adrenal glands of mice injected with SBC3-miR-1Zip-luciferase cells (Fig. 3F-G). Lung, liver, ovary, and adrenal glands are the most common metastatic sites for SCLC [33, 34]. Further, IHC staining analysis of SBC3/SBC3-miR-1Zip-xenografts tumors with proliferative and angiogenesis markers (Ki67 and CD31, respectively) revealed high Ki67 and CD31 staining in SBC3-miR-1Zip tumor tissues compared to SBC3 parental (Fig. 3H). These results suggest that miR-1 inhibition drives a highly metastatic SCLC phenotype in an intracardiac xenograft mouse model.

**Fig. 4:**
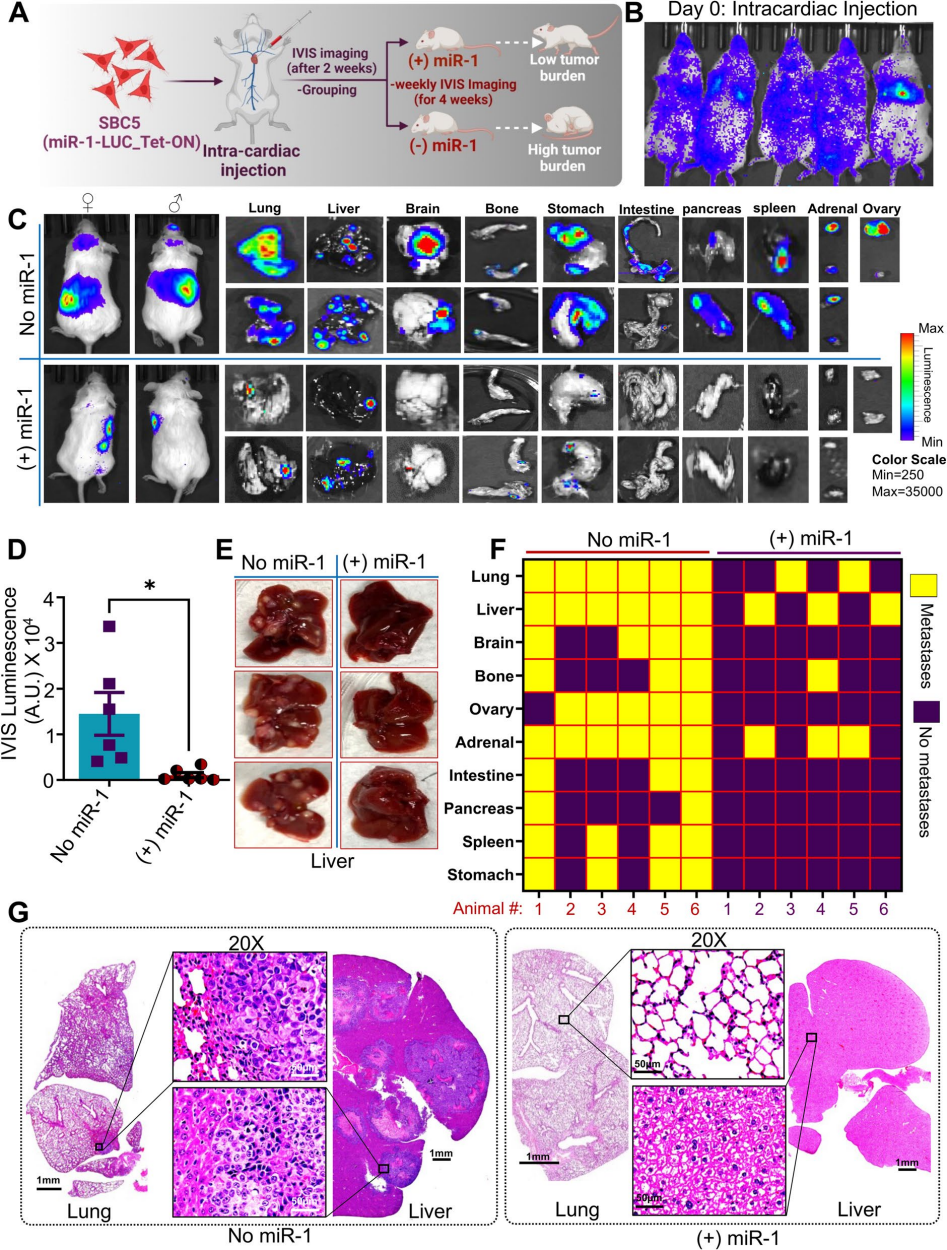
Ectopic overexpression of miR-1 decreases tumor growth and metastasis of intracardiac xenografts. **A** Schematic of SBC5 (-DOX-Off/ + DOX-On) intracardiac xenografts in NSG mice. **B** Representative IVIS images of NSG mice intracardially injected with SBC5 cells expressing luciferase (Day 0). **C** Representative IVIS images of mice bearing intracardiac xenografts on the end day of the experiment just before the time of sacrifice (one female and one male mice), with ex-vivo IVIS imaging of major organs showing SCLC metastasis such as lung, liver, brain, bone, stomach, intestine, pancreas, spleen, adrenal and ovary. Mice were injected with luciferin i.p. (150 mg/kg in sterile PBS) 5 min before euthanasia; following that the major organs were collected and scanned for metastatic lesions using IVIS imaging. **D** Quantification of the IVIS luminescence for each mice from no miR-1 and ( +)miR-1 group. **E** Representative *ex-vivo* images of liver showing metastasis nodules excised from no miR-1 and ( +)miR-1 groups. **F** Checkerboard representation of metastasis in the major organs of NSG mice as collected from SBC5 intracardiac xenografts of no miR-1(-DOX-Off) and + miR-1 (+ DOX-On) groups (data from *n* = 6 mice). Metastasis was studied using IVIS imaging and H&E analysis. **G** Representative H&E of liver and lung tissues having metastasis with an area highlighted with a box provided in higher-magnification or zoom inset. Scale bars represent 1 mm and 50 µm (with successive 20X magnification)

Next, to elucidate the impact of miR-1 overexpression on SCLC metastasis, we performed intracardiac injection of -DOX-off/DOX-On-miR-1 SBC5 cells (highly metastatic and relatively low miR-1 expression) in NSG mice (both male and female). Following two weeks of intracardiac injections, mice were randomized into ( +)miR-1/DOX-On and (-)miR-1/-DOX-off groups (Fig. 4A). The metastatic lesions were monitored using IVIS-imaging (Fig. 4B & C). Consistent with the metastatic phenotype of SBC5 cells, intracardiac xenografts showed substantial metastasis, with a high metastasis to most common SCLC metastatic sites, including, lung, liver, brain, bone, ovary, adrenal, stomach, intestine, pancreas, and spleen (Fig. 4C-F). On the contrary, exogenous miR-1 overexpression markedly decreased the metastatic potential and growth of intracardially injected SBC5 cells (Fig. 4C-F). In context to most frequent metastatic sites of SCLC including lung, liver, brain, and bone [33, 34], it was observed that the intracardiac injections of SBC5 cells mimicked the metastatic properties of SCLC and validated our metastatic model to study SCLC metastasis. The key observations from the metastasis studies, clearly showed that miR-1 has the potential to decrease SCLC metastasis at the most frequent metastatic sites of SCLC, such as lung, liver, brain, and bone (Fig. 4F). In addition to the primary metastatic sites of SCLC, the endocrine organs such as adrenal and ovary (in the case of female mice) were also reported as recurrent metastatic sites of SCLC [34–36]. The observation from metastasis studies suggests that the miR-1 overexpression decreased the adrenal and ovarian metastasis of SBC5 cells (Fig. 4 C & F). Interestingly, tissue histology studies using H&E staining further suggest that miR-1 overexpression decreased the tumor burden in the most frequent metastatic sites of SCLC such as lung, liver, and brain (Fig. 4G, supplementary Fig. S7-S8). Together, these findings suggest that miR-1 overexpression significantly inhibits SCLC growth and metastasis.

### miR-1 modulates the CXCR4/FOXM1/RRM2 axis in SCLC

Having established the antitumorigenic potential of miR-1 in SCLC, we next examined the molecular mechanism or molecules involved in the accomplishment of the tumor-suppressive role of miR-1. We first performed deep RNA sequencing (RNA-Seq) of SBC3, SBC3-miR-1Zip, SBC5(-DOX-off)/SBC5-DOX-On-miR-1 cells. Since SBC3 cells have low or basal expression of miR-1 and other cells have no miR-1 expression (Fig. 1H), we performed a differentially expressed gene (DEG) analysis in SBC3, SBC3-miR-1Zip, and SBC5 cells (supplementary Fig. S9). Upon comparative analysis of the top 50 DEG in SBC3, SBC3-miR-1Zip, and SBC5 cells, we found that *CXCR4* was the most differentially upregulated gene in SBC3-miR-1Zip and SBC5 cells (supplementary Fig. S9 & S10A).

**Fig. 5:**
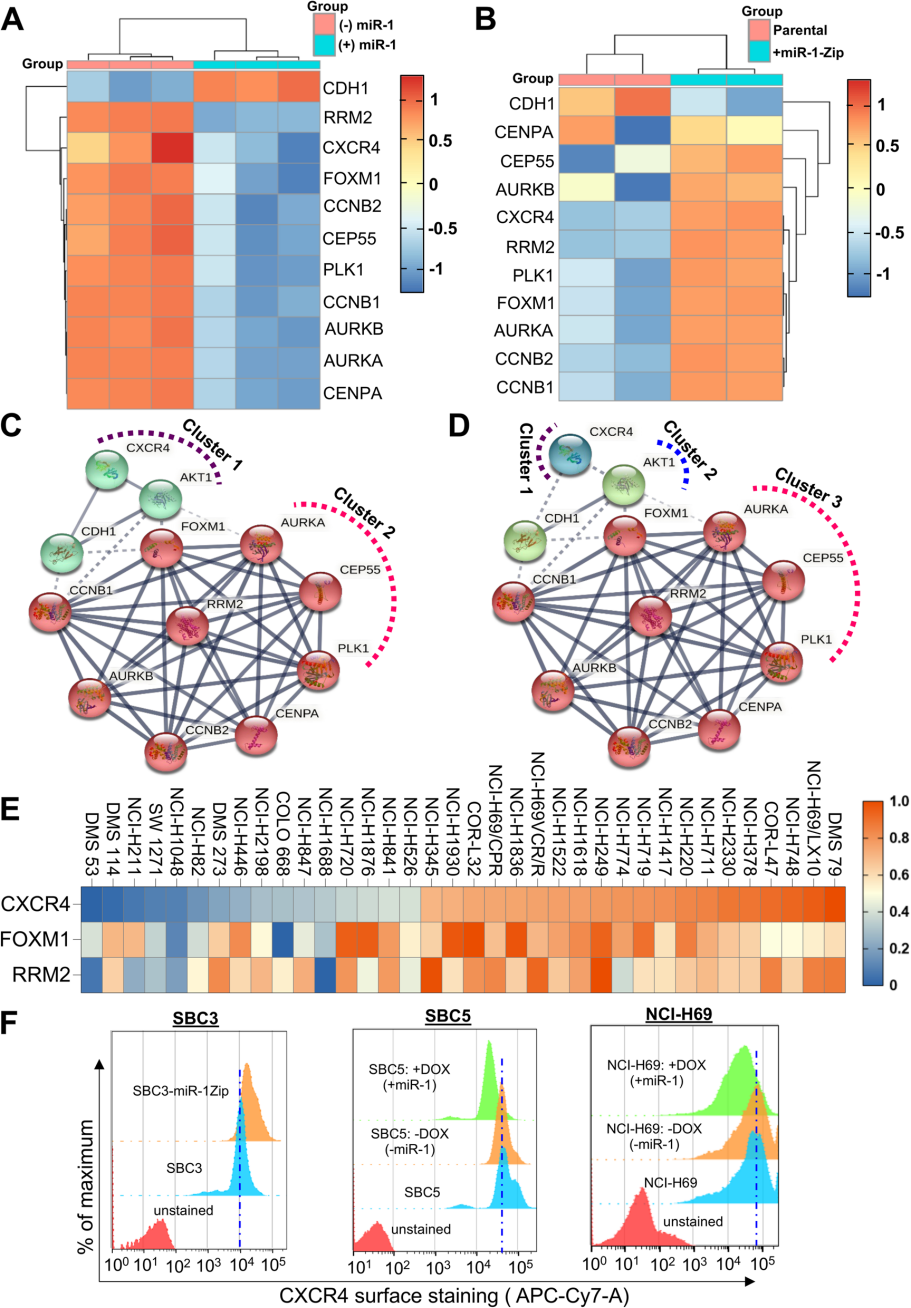
miR-1 modulates the CXCR4/FOXM1/RRM2 axis in SCLC. Heatmap of top differentially expressed genes from (**A**) miR-1(-DOX-Off) and + miR-1 (+ DOX-On) SBC5 cells, (**B**) SBC3 and SBC3-miR-1Zip cells. **C-D** Clustering and gene interaction network analysis (http://string-db.org/) of top differentially expressed genes from RNA-Seq analysis of miR-1 sponging and overexpression models. **E** Heatmap for normalized expression of CXCR4, FOXM1, and RRM2 in a panel of NCI SCLC cell lines data sets (https://discover.nci.nih.gov/rsconnect/SclcCellMinerCDB/). **F** Cell surface expression analysis of CXCR4 in SBC3, SBC3-miR-1Zip, SBC5, and NCI-H69 cells under no miR-1(-DOX-Off) and + miR-1 (+ DOX-On) conditions through FACS analysis

CXCR4 has been previously implicated in the growth and metastasis of various cancers, including SCLC [37, 38]. Previous studies have shown that CXCR4 mediates cancer cell adhesion, migration, chemoresistance, and metastasis, and small molecule or peptide inhibitors of CXCR4 such as AMD3100, TF14016, and LY2510924 decreased tumor growth and metastasis [39–41]. In the second set of RNA-seq analysis, we performed a comparative analysis of SBC5(-DOX-off)/SBC5-DOX-On-miR-1 and SBC3/SBC3-miR-1Zip cells to assess the DEG in the presence of high miR-1 (SBC5-DOX-On-miR-1) and sponged (miR-1Zip) or low miR-1 (Fig. 5 A-B). We observed a marked downregulation of SCLC-specific gene clusters (*CXCR4, RRM2, FOXM1, CCNB2, CEP55, PLK1, AURKA, AURKB, and CCNB1*) in SBC5 cells overexpressing miR-1 (Fig. 5A). Interestingly, we observed upregulation or enrichment of similar gene sets (*CXCR4, RRM2, FOXM1, CCNB2, CEP55, PLK1, AURKA, AURKB, CCNB1*) in SBC3-miR-1Zip cells compared to SBC3 (Fig. 5B). In addition, we observed marked enrichment of *CDH1* (gene encoding E-cadherin) in miR-1 overexpressing SBC5 cells, and consistent downregulation in SBC3-miR-1Zip cells (Fig. 5A-B).

Next, to investigate the tumor-suppressive mechanism of miR-1, we also performed clustering and interaction analysis of DEG (Fig. 5C-D). The clustering of DEG among miR-1 overexpressing and sponging groups suggested the existence of two to three clusters (Fig. 5C-D), solid lines connecting two members showed direct interaction within a cluster, whereas the dotted line showed the interaction between the members of two clusters. Clustering analysis showed that in the first cluster, *CXCR4* interacts with *AKT* or *CDH1*, and in the other cluster, *FOXM1* is represented as a central node for interaction with other DEG (Fig. 5C-D). FOXM1 has been previously implicated in SCLC tumorigenesis [42]. Thus, in addition to *CXCR4* (the candidate gene identified in the first set of RNA-seq analysis), the clustering analysis identified *FOXM1* as a second candidate gene co-operating with *CXCR4* and *RRM2,* a prominent downstream target of FOXM1 (Fig. 5C-D, supplementary Fig. S11-S13). RRM2 is known to regulate DNA-damage response, cancer aggressiveness, and drug resistance, and recent studies have validated FOXM1 as a RRM2-directing transcription factor [43, 44].

We further assessed the expression status of *CXCR4, FOXM1*, and *RRM2* in the SCLC cell line panel (SCLC-NCI, SCLC-UTSW, SCLC CCLE-Broad-MIT, SCLC GDSC-MGH-Sanger, SCLC CTRP-Broad-MIT, and SCLC Global) from the CellMiner-SCLC (https://discover.nci.nih.gov/SclcCellMinerCDB/) and observed a positive correlation in *CXCR4, FOXM1,* and *RRM2* expression (Fig. 5E, supplementary Fig. S11-S13). In most of the SCLC cell lines, high expression of *CXCR4, FOXM1,* and *RRM2* showing a positive correlation was observed (Fig. 5E, supplementary Fig. S11-S13).

**Fig. 6:**
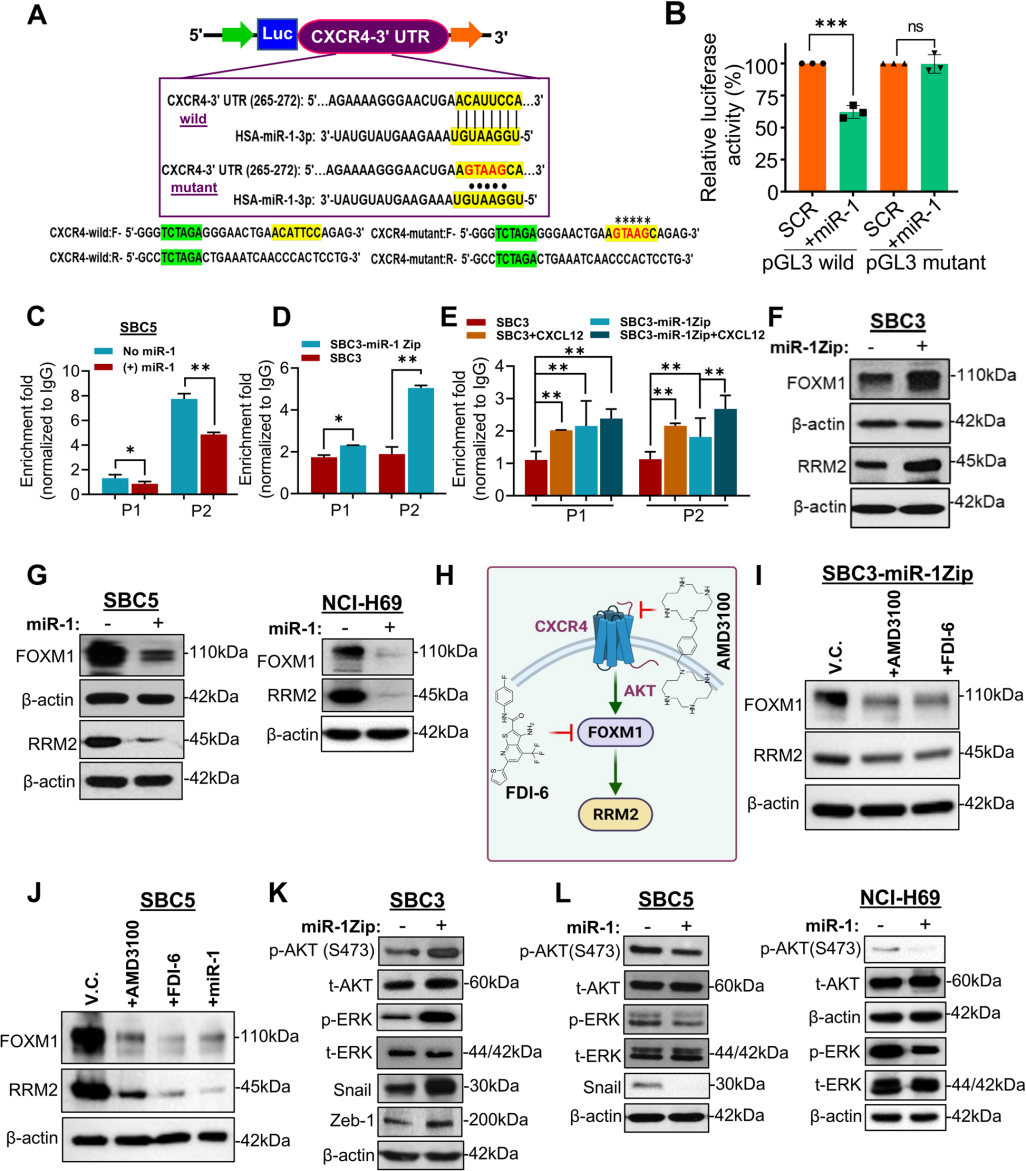
miR-1 targets CXCR4/FOXM1/RRM2, alters FOXM1 accessibility to RRM2 promoter, and modulates downstream signaling. **A** Schematic for the cloning of 3'-UTR sequences for dual luciferase assay. The upper panel of the inset shows the alignment of mature miR-1 sequences with 3'-UTR of CXCR4, and the lower panel shows the CXCR4 UTR residues mutated to abrogate the miR-1 binding. Primer sequences used to amplify 3'-UTR of CXCR4 were presented next to the inset. **B** Dual-luciferase assay validating CXCR4 as a target of miR-1. SBC5 cells were co-transfected with the luciferase-3'-UTR construct of CXCR4 (wild type/mutant), and miR-1 mimic, or scramble (SCR) and luciferase activity was measured. Chromatin immunoprecipitation of FOXM1 and RRM2 qPCR confirming the binding of FOXM1 with RRM2 promoter, (**C**) miR-1 decreased the interaction of FOXM1 with RRM2 promoter, (**D**) miR-1 sponging (miR-1Zip) enhances the accessibility of RRM2 promoter to FOXM1. **E** FOXM1 binding to RRM2 promoter in the presence of CXCL12 (100 ng/ml for 48-72 h), higher binding in miR-1Zip cells is due to high CXCR4 expression in these cells. **F** miR-1 sponging increased the expression of FOXM1 and RRM2 in SBC3 cells. **G** Immunoblotting studies showed that overexpression of miR-1 decreased the expression of FOXM1 and RRM2 in SBC5 and NCI-H69 cells. **H** Schematic to understand the viability of the CXCR4-FOXM1-RRM2 axis in SCLC cell lines. **I-J** SBC3-miR-1Zip and SBC5 cells were treated with AMD3100 (CXCR4 inhibitor) and FDI-6 (FOXM1 inhibitor) and analyzed for the expression of FOXM1 and RRM2 by Western blot. AMD3100 or FDI-6 treatment decreased the expression of FOXM1 and RRM2, like miR-1. Immunoblotting analysis demonstrated that; (**K**) miR-1 sponging increased the activation of AKT and ERK, and expression of epithelial to mesenchymal markers snail and Zeb-1 in SBC3 cells, (**L**) miR-1 overexpression decreased AKT and ERK activation in SBC5 and NCI-H69 cells. miR-1 also decreased the expression of snail in SBC5 cells. Statistical significance was calculated using two-tailed Student’s t-test for **C**-**D**, and one-way ANOVA for **E**. *, *p* < 0.05; **, *p* < 0.01; ***, *p* < 0.001; ns, non-significant

To better understand the transcriptional changes caused by miR-1 inhibition or overexpression, we performed protein expression studies by immunoblotting and IHC analyses of excised tumor tissues and the results further validated the overexpression of key targets observed in the RNA-seq/clustering analysis, including (1) high CXCR4 in SBC3-miR-1Zip tumor tissues compared to SBC3 parental tumor tissues (supplementary Fig. S14B), suggesting higher metastatic signaling that was confirmed in *in-vitro* CXCR4 surface expression analysis and oncogenic signaling (Figs. 5F, and 6); (2) high CXCR4 mediated signaling was also validated through CXCL12 treatment in SBC3 and SBC3-miR-1Zip cells, where a comparatively high colony formation, cell migration, and AKT activation was observed in CXCL12 treated SBC3-miR-1Zip cells compared to CXCL12 untreated or parental SBC3 cells (supplementary Fig. S5 C-F); (3) high levels of proliferation, EMT, and angiogenesis-related markers (Ki67, Zeb-1, snail, and CD31) in SBC3-miR-1Zip cells and tumor tissues (Figs. 3H and 6K); (4) increased expression of FOXM1 and RRM2 in SBC3-miR-1Zip cell lines and xenografts, which was validated in protein expression studies, and direct association of FOXM1 and RRM2 validated through a ChIP-assay (Fig. 6). In addition, high expression of miR-1 in normal lung tissues and low expression of miR-1 in SCLC tissues was validated through in situ hybridization, whereas low expression of CXCR4, FOXM1, and RRM2 in normal lung tissues and high expression of CXCR4, FOXM1, and RRM2 was observed in commercially available human SCLC tissue microarray (supplementary Fig. S15).

To explore the impact of miR-1 on the cell surface expression of CXCR4, we performed a flow cytometry-based cell surface expression analysis of CXCR4 in SCLC cells using APC-Cy-7-anti-CXCR4 (Fig. 5F). Remarkably, it was observed that miR-1Zip increased the cell surface expression of CXCR4 in SBC3 cells (Fig. 5F). On the other hand, exogenous expression of miR-1 using the DOX-ON-miR-1 system in SBC5 and NCI-H69 cells decreased the cell surface expression of CXCR4 (Fig. 5F). These experiments demonstrate that miR-1 decreases cell surface expression of CXCR4 in SCLC cell lines. Overall, our findings suggest that miR-1-mediated modulation of CXCR4 regulates oncogenic signaling and may drive the FOXM1-RRM2 axis in SCLC.

### miR-1 directly targets CXCR4 and alters FOXM1 accessibility to RRM2 promoter

We next sought to identify and characterize the miR-1 targeting site in the 3'-untranslated region (3'-UTR) of *CXCR4.* To this end, we retrieved and cloned the 3'-UTR of *CXCR4* that contained a miR-1 binding site (position 265–272 of 3'-UTR, identified using TargetScan) in the pGL3-luciferase vector *(for details, see* supplementary information*)*. In another set of 3'-UTR primers, we mutated the residues of the miR-1 binding site and cloned them into the pGL3-luciferase vector (Fig. 6A). A dual-luciferase assay was performed using miR-1 mimic with wild-type or mutant 3'-UTR-pGL3 constructs in SBC5 cells (as these cells have no miR-1) and found a decreased luciferase activity in SBC5 cells transfected with wild-type-3'-UTR-pGL3/miR-1 mimic compared to wild-type-3'-UTR-pGL3/scramble miR control (Fig. 6A-B). In contrast, no change in luciferase activity was observed in SBC5 cells transfected with mutant-3'-UTR-pGL3 /miR-1 mimic and mutant-3'-UTR-pGL3/scramble miR control (Fig. 6B).

To further analyze whether miR-1 directly modulates the *RRM2*-promotor targeting of FOXM1 or transcriptional regulation of RRM2 by FOXM1, we retrieved the FOXM1 binding sites in *RRM2* promoter from published ChIP-Sequencing data of FOXM1 and performed ChIP-coupled qPCR assay. Three pairs of primers (P1, P2, and P3) for FOXM1-binding sites in RRM2 promoter and a pair of negative control primers (10 kb upstream, non-FOXM1 binding region) were designed to study the FOXM1 binding in SCLC cell lines under different experimental conditions (*for details see* supplementary information). We found that miR-1 overexpression in SBC5 cells (DOX-On-miR-1 SBC5) decreased the enrichment of FOXM1 with RRM2-promoter (Fig. 6C), whereas an enhanced binding or enrichment of FOXM1 to the *RRM2*-promoter has been observed in SBC3-miR-1Zip cells compared to SBC3 parental cells (Fig. 6D). Further, to validate the dependency of FOXM1 binding to RRM2 promoter with miR-1/CXCR4 axis, we also performed ChIP-qPCR in SBC3-parental, SBC3-miR-1Zip, SBC5, and DOX-On-miR-1 SBC5 cells in the presence of CXCL12 (CXCR4 ligand). Interestingly, a significant increase in the FOXM1-binding was observed with RRM2 promoter in SBC3 parental and SBC3-miR-1-Zip cells in CXCL12 treated vs. non-treated cells (Fig. 6E). In addition, CXCL12 treatment increased the expression of FOXM1 and RRM2 in SBC3 cells (Fig. S16A). On the other hand, CXCL12 treatment in the presence of miR-1 did not affect the FOXM1-binding with RRM2 promoter in SBC5 cells (Fig. S16B). Altogether, the CXCR4 cell surface expression analysis (Fig. 5F), 3'-UTR targeting, and FOXM1-RRM2 ChIP-qPCR experiments demonstrate that miR-1 targets CXCR4 and significantly decreases the accessibility of FOXM1 to RRM2-promoter (transcriptionally decreased RRM2), implying that miR-1 targets the CXCR4/FOXM1-RRM2 axis in SCLC.

### miR-1 targets the CXCR4/FOXM1/RRM2 axis, and pharmacological inhibition of CXCR4 and FOXM1 phenocopy miR-1 efficacies

Next, we aimed to validate our transcriptional findings in SCLC cell line models and xenograft tumor tissues through protein expression studies. Consistent with the RNA-Seq data, it was observed that miR-1 sponging increased the expression of FOXM1 and RRM2 in SBC3 cells compared to parental cells (Fig. 6F). Interestingly, the ectopic expression of miR-1 in DOX-On-miR-1 -SBC5 and/or -NCI-H69 cells consistently decreased the expression of FOXM1 and RRM2 (Fig. 6G). In concordance with CXCR4 cell surface expression studies (Fig. 5F), these observations suggested that miR-1 targets the CXCR4/FOXM1/RRM2 axis in SCLC.

To further establish whether the CXCR4/FOXM1/RRM2 axis is a viable target of miR-1, we took advantage of AMD3100 and FDI-6, the well-characterized and highly specific small molecule inhibitors of CXCR4 and FOXM1, respectively [45, 46]. The proposed model of utilizing AMD3100 and FDI-6 has been shown in Fig. 6H. As expected, it was found that AMD3100 treatment resulted in decreased expression of FOXM1 and RRM2 in SBC3 and SBC3-miR-1Zip cells, and treatment of cells with FDI-6 also decreased the expression of FOXM1 and RRM2 (Fig. 6I, supplementary Fig. S17A). We followed the treatment of AMD3100 and FDI-6 in SBC5 cells and compared the expression profile of FOXM1 and RRM2 in these cells with that of SBC5 cells that ectopically overexpressed miR-1 (DOX-On-miR-1 -SBC5). Remarkably, it was observed that the treatment of SBC5 cells with either AMD3100 or FDI-6 decreased the expression of FOXM1 and RRM2, and phenocopy the consequence of miR-1 overexpression concerning FOXM1 and RRM2 expression (Fig. 6J). Additionally, to validate the relationship of the FOXM1/RRM2 axis in SCLC cells, we performed genetic perturbation of *FOXM1* using small interfering RNA (siRNA)-mediated knockdown in SBC5 cells and found that knockdown of *FOXM1* decreased the expression of RRM2 (supplementary Fig. S17B). Furthermore, we have analyzed the expression or activation of survival and metastasis-related markers (p-AKT/AKT, p-ERK/ERK, Snail, and Zeb-1), and observed that miR-1 inhibition enhances the activation of AKT and ERK and increases the expression of snail and Zeb-1 in SBC3 cells (Fig. 6K). The overexpression of miR-1 in SBC5 and NCI-H69 cells decreases the expression of snail, and the activation of AKT and ERK (Fig. 6L). Collectively, these results suggest that miR-1 modulates the CXCR4/FOXM1/RRM2 axis in SCLC, and ectopic expression of miR-1 inhibits the expression of metastasis-associated proteins (snail) and activation of AKT and ERK.

**Fig. 7:**
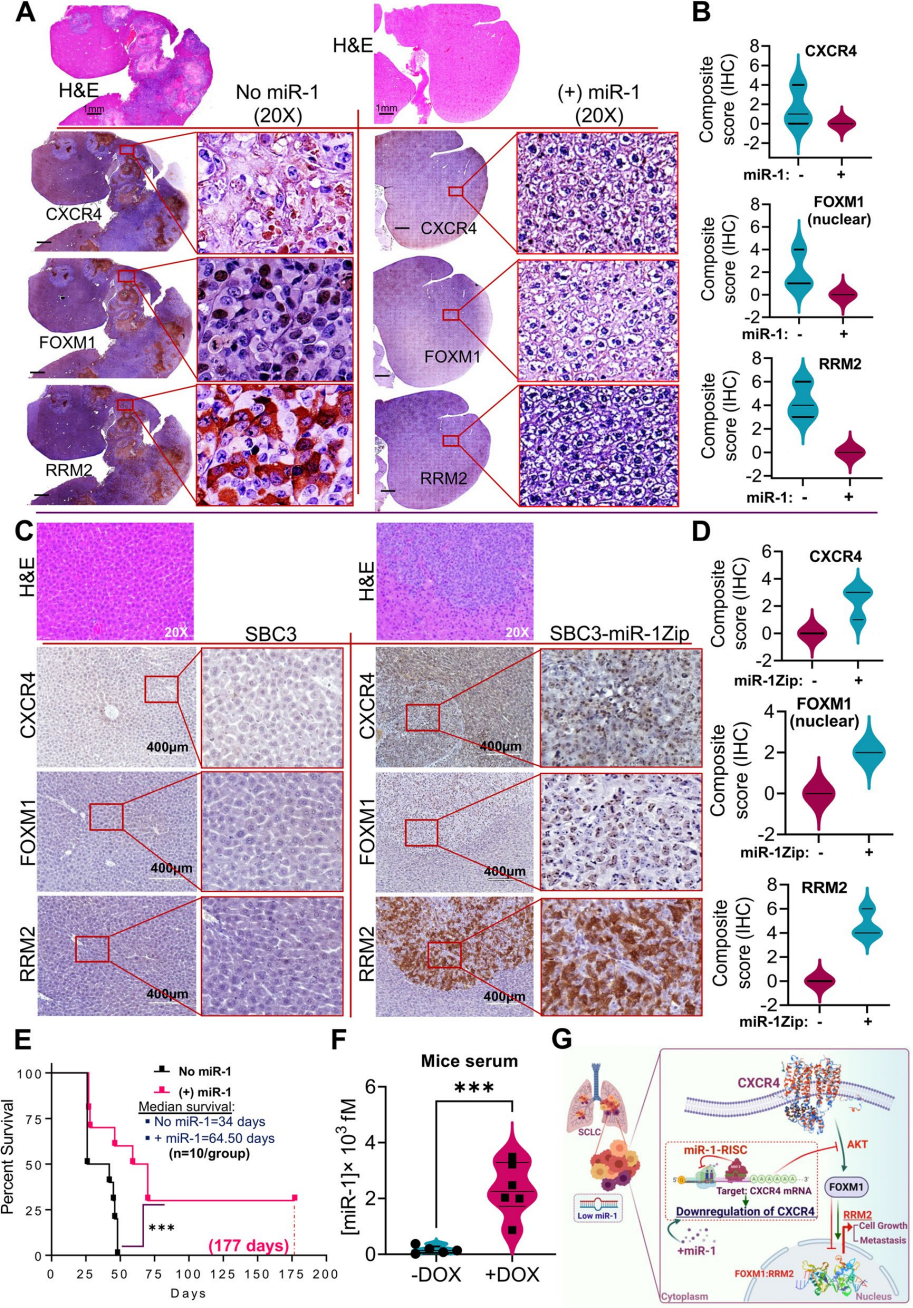
miR-1 modulates the CXCR4/FOXM1/RRM2 axis and enhances survival. **A** Representative H&E and IHC of serial sections of same liver tissue having metastasis for CXCR4, FOXM1, and RRM2 from SBC5 no miR-1(-DOX-Off) and + miR-1 (+ DOX-On) intracardiac xenografts. Image of whole liver tissue sections having metastasis with an area highlighted with a box provided in higher-magnification or zoom inset. Scale bars represent 1 mm (with successive magnification). **B** IHC quantification for respective proteins was presented in the right panels (*n* = 6 liver sections). **C** Representative H&E and IHC of the same liver tissue having metastasis for CXCR4, FOXM1, and RRM2 from intracardiac xenografts of SBC3 and SBC3-miR-1Zip cells. Scale bars represent 400 µm (with successive magnification). **D** IHC quantification for respective proteins was presented in the right panels (*n* = 6 liver sections). **E** Kaplan–Meier survival analysis of NSG mice bearing metastatic SCLC xenografts under no miR-1 and + miR-1 conditions for 24 weeks (*n* = 10 mice in each group). **F** Violin with point graph representing the quantitative expression of serum miR-1 in -DOX-Off/ + DOX-On group of NSG mice. Statistical significance was calculated using two-tailed Student’s t-test for unpaired samples, where ***, *p* < 0.001. **G** Overall postulated mechanism of miR-1 mediated attenuation of SCLC cell growth and metastasis. Low miR-1 and high CXCR4 were associated with SCLC and proposed therapeutic activation or overexpression of miR-1 in SCLC target 3'-UTR of *CXCR4,* resulting in decreased CXCR4 expression. In addition, miR-1 mediated CXCR4 targeting inhibited FOXM1/RRM2 axis responsible for SCLC growth and metastasis. Schematic created with BioRender.com

To determine whether the CXCR4/FOXM1/RRM2 axis or the expression pattern that we observed in RNA-Seq and in vitro SCLC models follow a similar pattern in intracardiac xenografts in vivo models or not, we performed expression analysis of CXCR4, FOXM1, and RRM2 using IHC on metastatic liver tissues (as the liver is the most frequent metastatic site of SCLC). Metastatic liver tumors revealed a high expression of CXCR4, FOXM1, and RRM2 in the tissues excised from the no-miR-1 group (SBC5-DOX-off) of mice, whereas the majority of liver tissue sections from the + miR-1 group (SBC5-DOX-On-miR-1) of mice showed very low to no expression of CXCR4, FOXM1, and RRM2 (Fig. 7A). The pathological quantification of liver tissues stained with CXCR4, FOXM1, and RRM2 further confirmed that the expression of these markers significantly decreased in the + miR-1 group (Fig. 7B). We repeated the IHC staining of CXCR4, FOXM1, and RRM2 in the liver tissues from SBC3 and SBC3-miR-1Zip intracardiac xenografts and observed intense staining of these proteins at the liver metastatic sites developed from SBC3-miR-1Zip cells, and in contrast, no staining was observed in the SBC3 injected group (Fig. 7C-D). Taken together, the IHC-analysis supports that miR-1 directs the CXCR4/FOXM1/RRM2 axis in SCLC.

### SCLC xenografts derive greater survival benefits from high miR-1

Given that miR-1 loss aggravates SCLC growth and metastasis, whereas exogenous overexpression of miR-1 decreases the metastatic proficiencies of SCLC cells (Figs. 3 and 4), we next asked whether miR-1 has any effect on the overall survival of mice bearing SCLC tumors. Therefore, to explore the clinical implications of miR-1 in terms of survival, we again performed intracardiac injections of DOX-On-miR-1 SBC5 cells in NSG mice and scrutinized for the establishment of metastasis using IVIS imaging. The animals were randomized into ( +)miR-1/ + DOX and (-)miR-1/-DOX groups. Strikingly, miR-1 expression significantly prolonged the survival of mice standing intracardiac SCLC xenografts compared to the no miR-1 group (Fig. 7E). The estimated median survival was nearly 65 days for the (+) miR-1 group, whereas it was only 34 days for (-)miR-1 group (Fig. 7E). Next, we explored the possibility of detecting miR-1 levels in the serum of mice intracardially injected with SCLC cells using the DNA-AuNPs nanoprobe-mediated detection assay we developed (Fig. 1E). Interestingly, a significantly high miR-1 level was observed in the serum samples of mice showing prolonged survival (Fig. 7F). Collectively, this data show that miR-1 provided survival benefits in vivo as it promotes the survival of mice injected with SCLC cells and decreased tumorigenesis and metastasis.

## Discussion

SCLC is one of the most aggressive types of cancer with proven challenges of limited therapeutic options. Based on its coherent histology, SCLC was classically considered a homogenous tumor and remained a ‘poster child’ disease; however, recent studies involving the compilation of high throughput transcriptomic approaches established that SCLC represents a highly dynamic cancer type with high levels of subtype switching and plasticity [47–49]. In comparison with other cancers, the DNA and RNA sequencing studies of SCLC tumors identified a limited number of actionable therapeutic targets or molecules that may be applied to patients, and owing to high neuroendocrine (NE) and/or non-NE intratumoral plasticity, these limited options present a challenge in the generation of personalized therapies. Thus, there remains an imperative need to identify novel therapeutic molecules or targets that can improve SCLC outcomes.

In this study, using bioinformatics analysis of publicly available datasets, human tissues, cell lines, and serum analysis, we identified miR-1 as an important tumor suppressor gene in SCLC. The mature single *MiR-1* is encoded by two separate genes, *MIR-1–1* and *MIR-1–2* located on separate chromosomal loci (20q13.33 and 18q11.2, respectively) [17, 19]. The role of miR-1 has been well described in cardiomyocytes and skeletal muscle precursor cells [17]. It regulates the differentiation of smooth muscle cells, innate immune response, glucose-mediated apoptosis, and proliferation and invasion of gastric cancer cells through transforming growth factor-beta signaling [50–52]. Interestingly, the serum signature of miR-1 was reported as a prognostic factor in NSCLC [53]. Using a noninvasive serum transcriptomic-based and nanoprobe-mediated detection system for miR-1, we find low serum miR-1 in SCLC patients compared to healthy donors and simultaneously observed low or null miR-1 expression in human SCLC tumor tissues compared to normal lung tissues. In addition, our in vivo studies showed a survival benefit in the mice bearing intracardiac xenografts having high miR-1 levels. The median survival of mice with high miR-1 is nearly two-fold higher than the low miR-1 group, thus, identifying low miR-1 as a hallmark of SCLC.

Of interest, SCLC is considered a metastatic disease, accounting for ~ 70% of patients who show distant metastasis at the time of diagnosis [34]. CXCR4 is a well-characterized chemokine receptor that regulates cancer metastasis, including SCLC metastasis [37–40]. The inhibition of CXCR4 using small molecule inhibitors suppressed SCLC metastasis in vivo [39, 54]. Interestingly, we observed that miR-1 directly targets 3'-UTR of CXCR4 in SCLC cell lines. Our mechanistic studies revealed that miR-1 modulates CXCR4 expression in SCLC cells and metastatic lesions of intracardiac xenografts. Our data also suggest that miR-1 mediated CXCR4 modulations require FOXM1 pathway activity to promote tumor growth and metastasis. Our observations from miR-1 overexpression or knockdown cell line models suggest that miR-1 targets the CXCR4/FOXM1 axis; therefore, it is critical that we better define miR-1 modulatory effects on CXCR4/FOXM1 axis. Moreover, we confirmed these results with AMD3100, a highly specific small-molecule inhibitor of CXCR4. Inhibition of CXCR4 using AMD3100 decreased the expression of FOXM1, demonstrating that FOXM1 is downstream of CXCR4 inhibition that results in FOXM1 suppression.

FOXM1 is a transcription factor that belongs to the Forkhead superfamily, with a highly conserved (winged helix) DNA-binding domain and plays an essential role in the regulation of a plethora of biological mechanisms, including cell cycle regulation, angiogenesis, cell proliferation, DNA damage repair, and apoptosis [55, 56]. Studies suggest that tumor suppressor p53 is required for FOXM1 downregulation that follows a retinoblastoma (Rb) dependent mechanism [57]. In context to frequent deletion or loss of function mutations in *TP53* and *RB1* [7, 8], and consistent with a recent report that shows a crucial role of FOXM1 using *Rb1*^*fl/fl*^*; Trp53*^*fl/fl*^*; Lox-Stop-Lox [LSL]-Myc*^*T58A*^ (RPM) models and patient samples in SCLC tumorigenesis and poor clinical prognosis [42], our work builds an agreement that miR-1 mediated downregulation of CXCR4/FOXM1 axis could be a major event regulating SCLC growth and metastasis. In addition, two independent studies in SCLC suggest that high FOXM1 and CXCR4 decrease the overall survival of SCLC patients compared to the low FOXM1/CXCR4 group [42, 58]. Consistent with the previous studies that showed FOXM1 is regulated in a p53 and Rb/E2F dependent manner, our data suggest that miR-1 overexpression downregulated SCLC specific gene signatures such as *CXCR4, FOXM1, PLK1, CENPA, AURKA/B,* and *RRM2* [57, 59, 60], while miR-1 inactivation increased their expression*.* Interestingly, most of the differentially regulated genes in miR-1 overexpression system contribute to the progression of SCLC; however, RRM2 is one of the top differentially regulated targets (among top 15 DEG), therefore on the basis of interactome analysis and GSE analysis (in miR-1 overexpression/knockdown system), we selected RRM2 as a prominent target of CXCR4/FOXM1 axis.

Notably, we observed that miR-1 overexpression decreased the expression of RRM2, while miR-1 sponging increased RRM2 expression. RRM2 is a reductase that catalyzes the conversion of ribonucleotides to deoxyribonucleotides, protects cancer cells from replication stress, induces drug resistance, regulates purine metabolism, cell cycle, and DNA repair [61, 62]. Our unbiased RNA-Seq and clustering data identified *RRM2* as a major target gene regulated by FOXM1 in SCLC. Using genetic and pharmacological tools, we demonstrated that inhibition of FOXM1 decreases RRM2 expression in SCLC cell lines. We envisage that in SCLC cells, FOXM1 might have direct interactions with the promoter region of RRM2. Our ChIP assay established that FOXM1 has an affinity for the promoter region of *RRM2* and miR-1 modulates the binding of FOXM1 with the RRM2 promoter. In addition, the FOXM1 ChIP analysis in the presence of CXCL12 (CXCR4 ligand) and miR-1 knockdown suggested enhanced FOXM1 binding to the promoter region of *RRM2*, further supporting CXCR4-mediated modulation of FOXM1-RRM2 interactions in SCLC. An interesting study has demonstrated recently that FOXM1 transcriptionally activates RRM2 in prostate cancer [43]. RRM2 has been implicated in epithelial to mesenchymal transition (EMT) and tumor-associated angiogenesis [58, 59]. Consistent with this, we observed a significant downregulation of EMT markers (Zeb-1, Snail) in miR-1 overexpressing SCLC cell lines, and upregulation of these EMT markers along with CD31 (a marker of angiogenesis) had been observed in miR-1 knockdown conditions. In transcriptomics studies, we found the enrichment of CDH1 (E-cadherin) in miR-1 overexpressing cell lines, whereas miR-1 knockdown was coupled with a decrement in CDH1. Taken together, the outcomes of the gene enrichment study, ChIP-assay, and pharmacological inhibition of CXCR4 or FOXM1, or genetic knockdown of FOXM1 establish that miR-1 modulates the CXCR4/FOXM1/RRM2 axis in SCLC cells (Fig. 7G).

Progressively, recent studies are identifying subtype-specific unique and viable therapeutic vulnerabilities for SCLC; for example, NOTCH activation, inhibition of AURKA/B, LSD1, HDAC, BET, and EZH2 [63–67]. Our work builds an interesting concept where it implicates the outcomes of the present study to develop a potential therapeutic window for the activation/overexpression of miR-1 to inhibit SCLC or targeted delivery of miR-1 using alternative approaches such as nanoparticle-mediated delivery of small RNA/DNA molecules. The utilization of miRNAs as inhibitors or activators is still in the developing stages and needs to be further improved or developed to cope with the undesired or off-target effects; however, our efforts also highlight the possibilities of targeting the CXCR4/FOXM1/RRM2 axis to thwart SCLC growth and metastasis (Fig. 7G).

## Conclusions

In summary, our findings reveal that miR-1 is a critical regulator for SCLC growth and metastasis. Owing to the antitumorigenic role of miR-1 in SCLC, our results suggest a novel mechanistic insight that miR-1 targets the CXCR4/FOXM1/RRM2 axis that attenuates SCLC growth and metastasis. This study sheds light on the potential role of miR-1 in SCLC and rationalizes the therapeutic targeting of the CXCR4/FOXM1/RRM2 axis or individual members of this axis for the development of novel SCLC therapies. In addition, we have designed a highly sensitive nanoprobe-mediated noninvasive miR-1 detection method that could be utilized as a future diagnostic tool for miR-1 or can be optimized for detecting other miRNAs. These findings further provide a platform for utilizing serum miR-1 levels as a potential biomarker or noninvasive predictor for the survival and prognosis of this devastating disease.

## Supplementary Information

**Additional file 1.**

**Additional file 2.**

## Data Availability

The data supporting the results and conclusions of this article are included in this article and its supplementary files. The RNA-Sequencing data generated in this study were deposited to NCBI SRA repository with accession number PRJNA900568 and will be available publicly following the publication of this article.

## Abbreviations

NSCLC: Non-small cell lung cancer
SCLC: Small cell lung cancer
RNAi: RNA interference
miRNAs: MicroRNAs
CDH1: Cadherin 1
RISC: RNA-induced silencing complex
miR-1: MicroRNA-1-3p
CXCR4: C-X-C motif chemokine receptor 4
FOXM1: Forkhead box protein M1
DEG: Differentially expressed genes
RRM2: Ribonucleotide reductase regulatory subunit M2
TEM: Transmission electron microscopy
ISH: In situ hybridization
DNA-AuNPs: DNA-probe-modified Au-nanoparticles
