# Supplementary material for: MicroRNA-1 attenuates the growth and metastasis of small cell lung cancer through CXCR4/FOXM1/RRM2 axis
Source: Mol Cancer. 2023 Jan 4;22:1. doi: 10.1186/s12943-022-01695-6 (PMC9811802; doi:10.1186/s12943-022-01695-6)
Supplement: Supplementary file 1 — Additional file 1. [file 12943_2022_1695_MOESM1_ESM.pdf]

Supplementary Figure S1

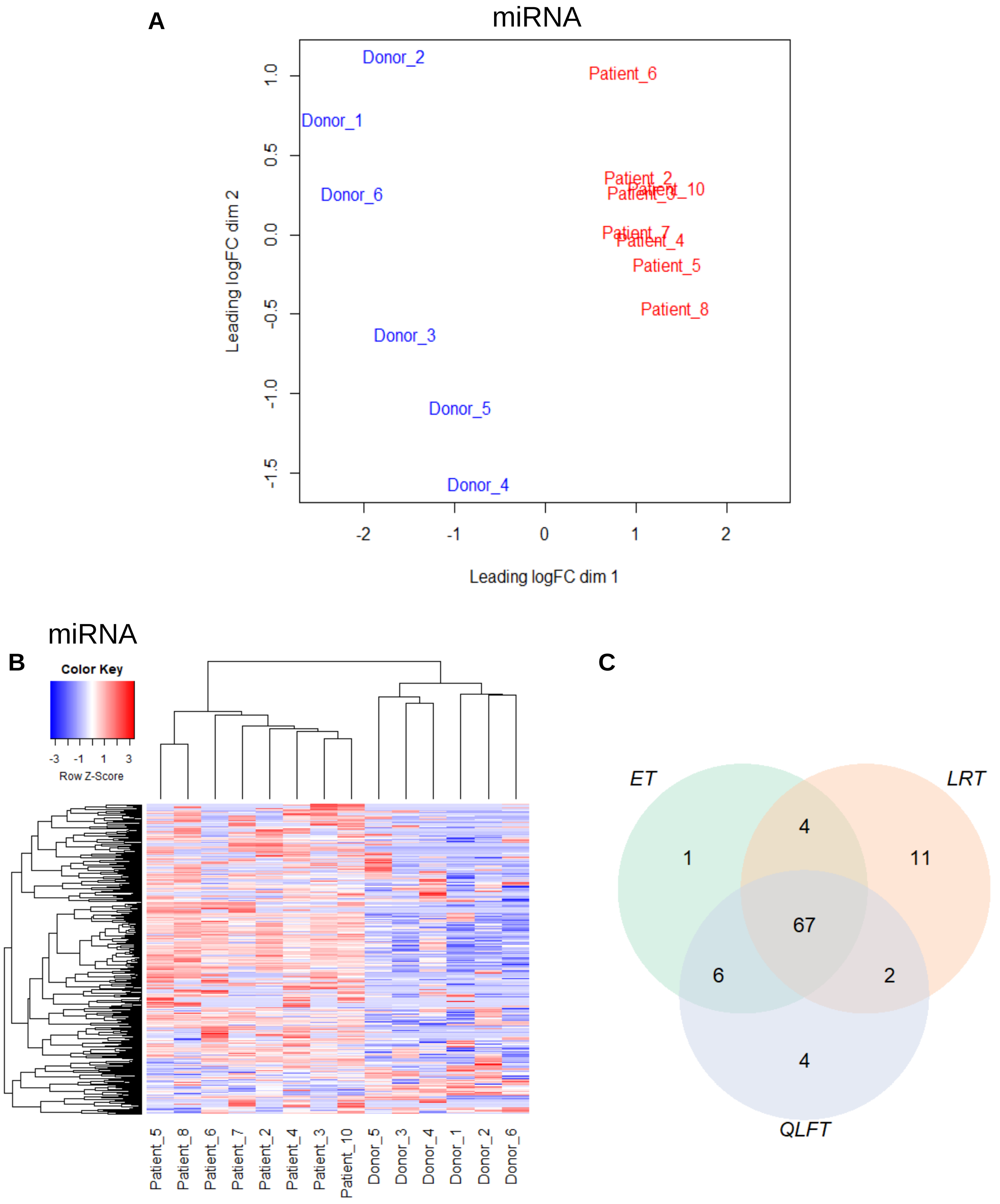

Figure S1: MiRNA sequencing analysis from serum samples of SCLC patients and healthy donors. (A) MDS plot: Multidimensional scaling plot of distances between gene expression profiles (before filtering low expression genes). **(B)** Heatmaps showing hierarchical clustering after filtering low expression genes (requiring at least three samples satisfying cpm>1). 462 miRNA's (2126 filtered). **(C)** edgeR was applied for differential expression (DE) analysis. Three statistical methods were compared to determine DE genes; Exact Test (ET), Likelihood Ratio Test (LRT), and Quasi-likelihood F-test (QLFT).

Supplementary Figure S2

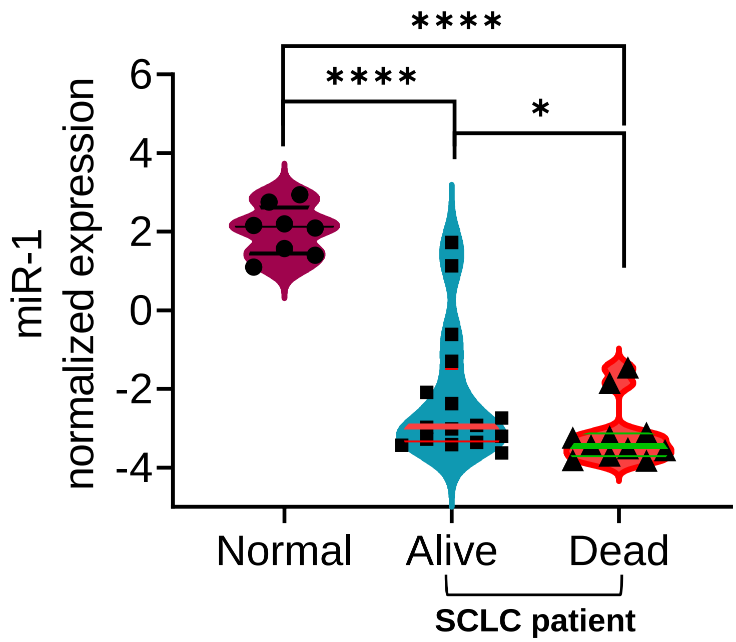

Figure S2: Normalized expression of miR-1 in Violin plots comparing the expression profile of miR-1 between normal lung tissues (n=8) and SCLC tumor tissues from the patients, alive (n=16) and dead (n=11) in the publicly available gene expression data of human cancers (GSE19945). Statistical significance was considered using one-way ANOVA and t-test. *, $p<0.05$; ****, $p<0.0001$.

Supplementary Figure S3

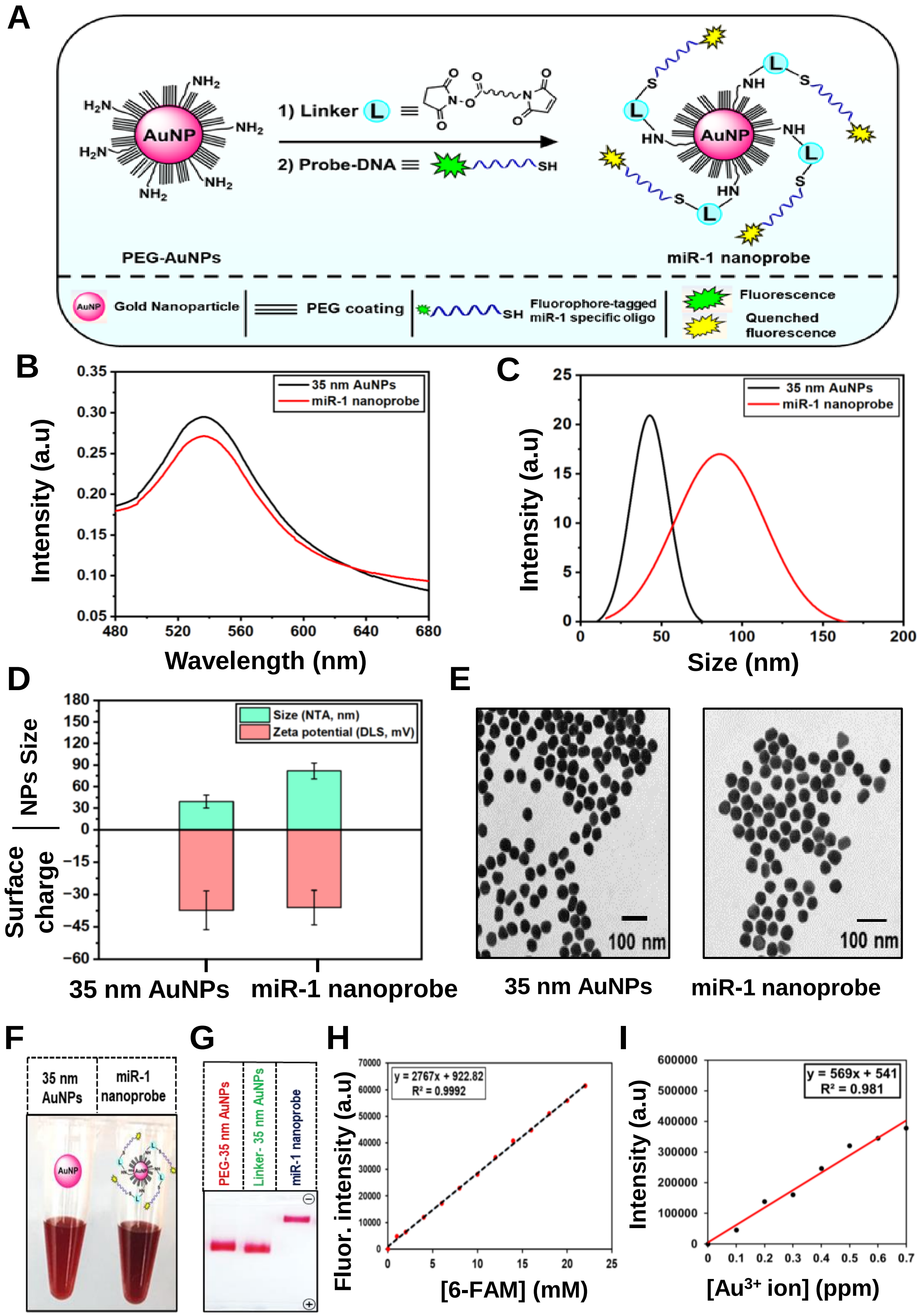

Figure S3: (A) Representative scheme for the synthesis of DNA-AuNPs by conjugating DNA-probes to the surface of the PEG-passivated AuNPs. **(B)** UV-vis spectra, **(C)** DLS spectra, **(D)** hydrodynamic diameter (calculated by NTA) and zeta potential (calculated by DLS) of nm **(E)** TEM images (scale bar, 100 nm) of the 35 nm AuNPs and DNA-AuNPs. **(F)** Agarose gel electrophoresis analysis (2% gel photograph) of before and after DNA-probe functionalization on AuNPs. **(G)** A standard calibration curve (from fluorescence assay and ICP-OES) was plotted for the calculation of quantification of No. of DNA-probes per AuNPs (N_{Oligo}).

Supplementary Figure S4

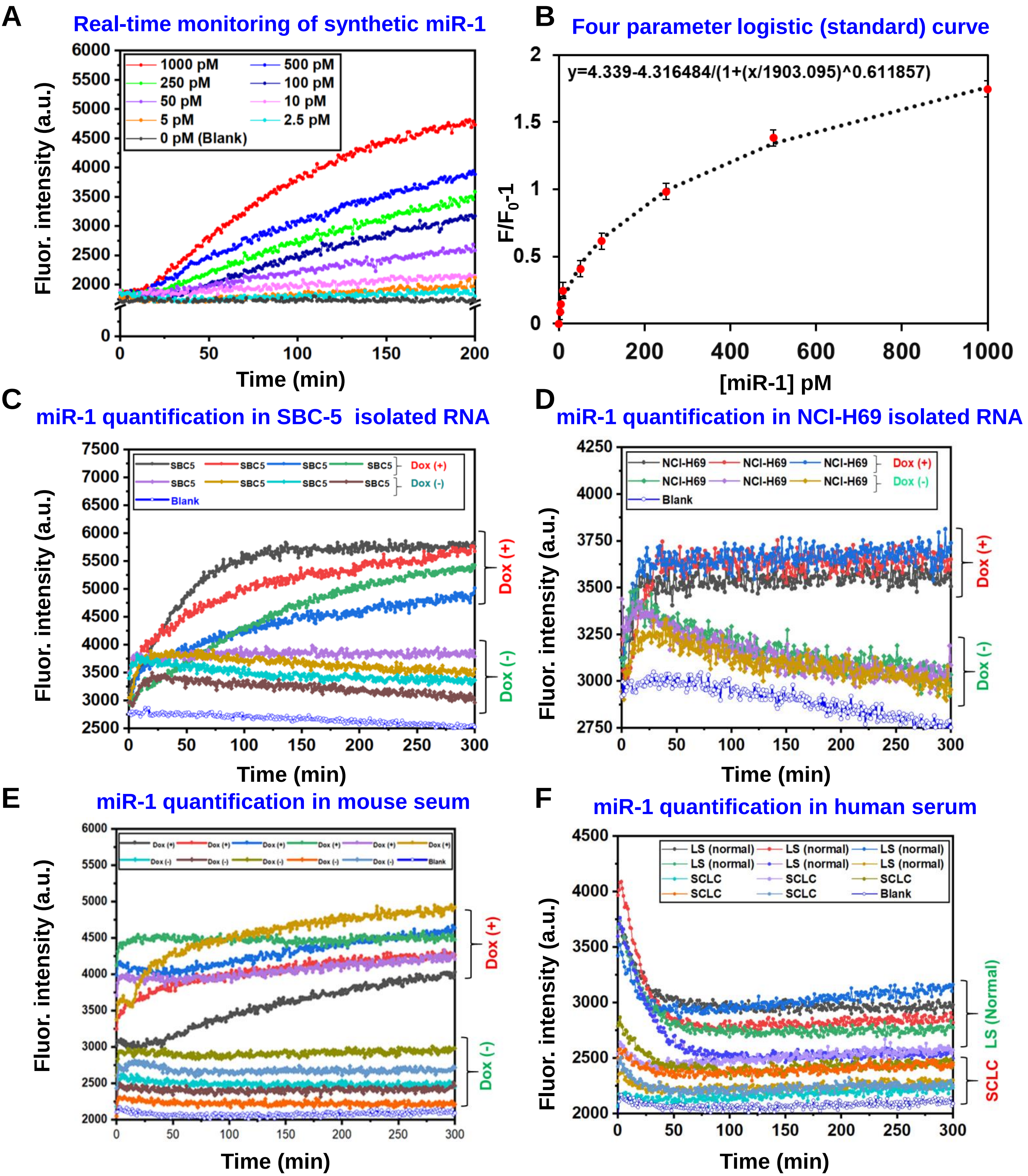

Figure S4: (A) Real-time monitoring of variable concentrations of synthetic miR-1 target. **(B)** Calibration curve establishment: Linear signal response between fluorescence ratio (F/F_0 -1) and the sequentially increased amounts of miR-1. Representative raw data (fluorescence intensity vs. time) showing detection of miR-1 from total RNA isolated from **(C)** SBC-5 and **(D)** NCI-H69 cells after treatment with Doxorubicin. The *in situ* detection of miR-1 in **(E)** SCLC mice vs. control mice serum. **(F)** Human SCLC patient vs. normal control serum.

Supplementary Figure S5

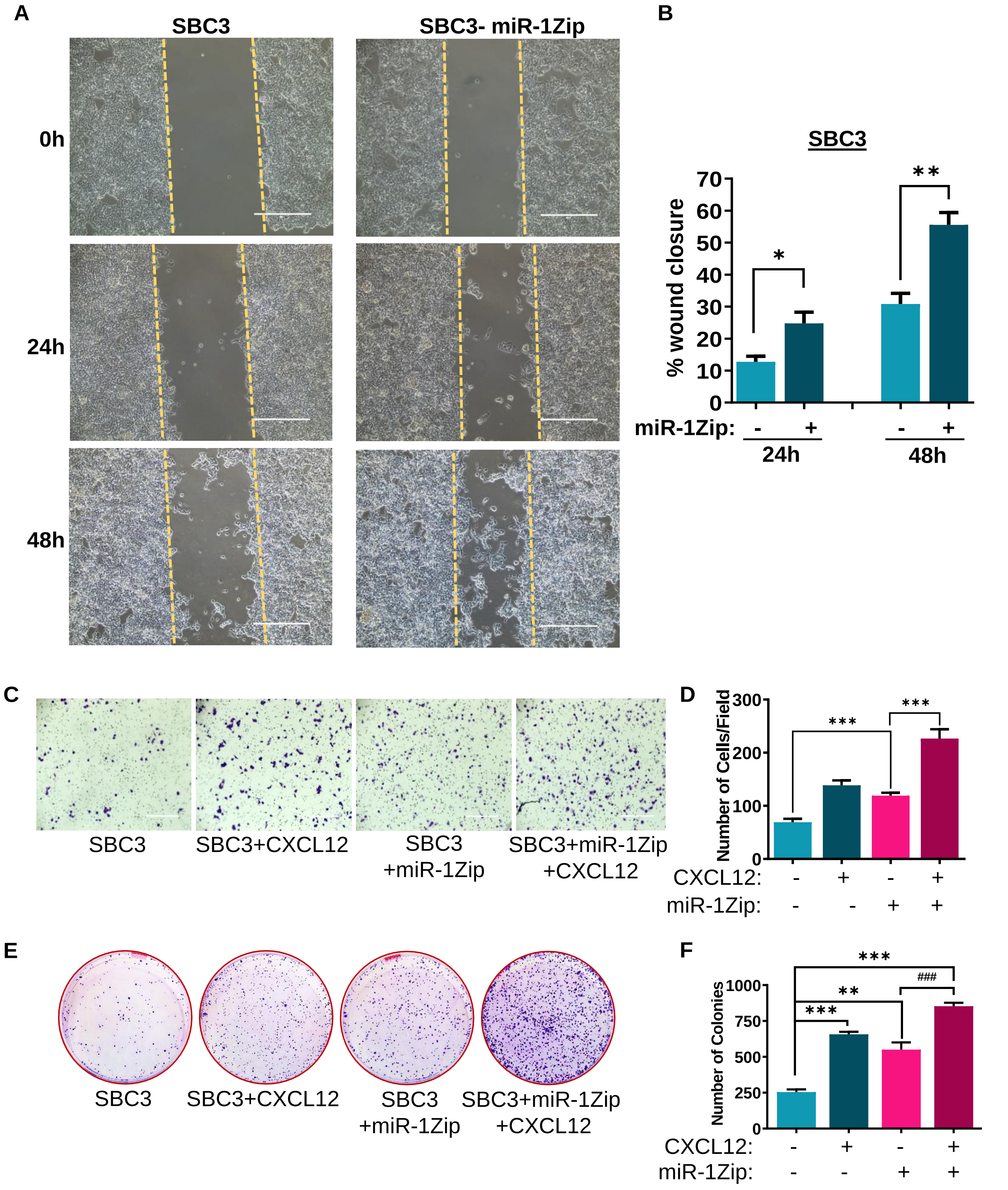

Figure S5: Stable knockdown of miR-1 increases cell migration or CXCL12-induced cell migration and colony formation in SCLC cells. (A) SBC3 (control) and miR-1 knockdown (SBC3-miRZip) were seeded in 6-well plate, an artificial wound was created using 200 µl pipet tip and washed with PBS and observed for cell migration (wound healing). Photomicrographs were taken at 10X imaging areas of the wounds on 0h, 24h and 48h. **(B)** Quantification of percent wound closure in wound healing assay. SBC3 (control) and miR-1 knockdown (SBC3-miRZip) cells in the presence or absence of CXCL12 (100 ng/ml) as indicated were subjected for **(C)** transwell cell migration assay, **(D)** Quantification of the number of migrated cells, **(E)** colony formation assay **(F)** Quantification of the number of colonies under different experimental conditions.,

Supplementary Figure S6

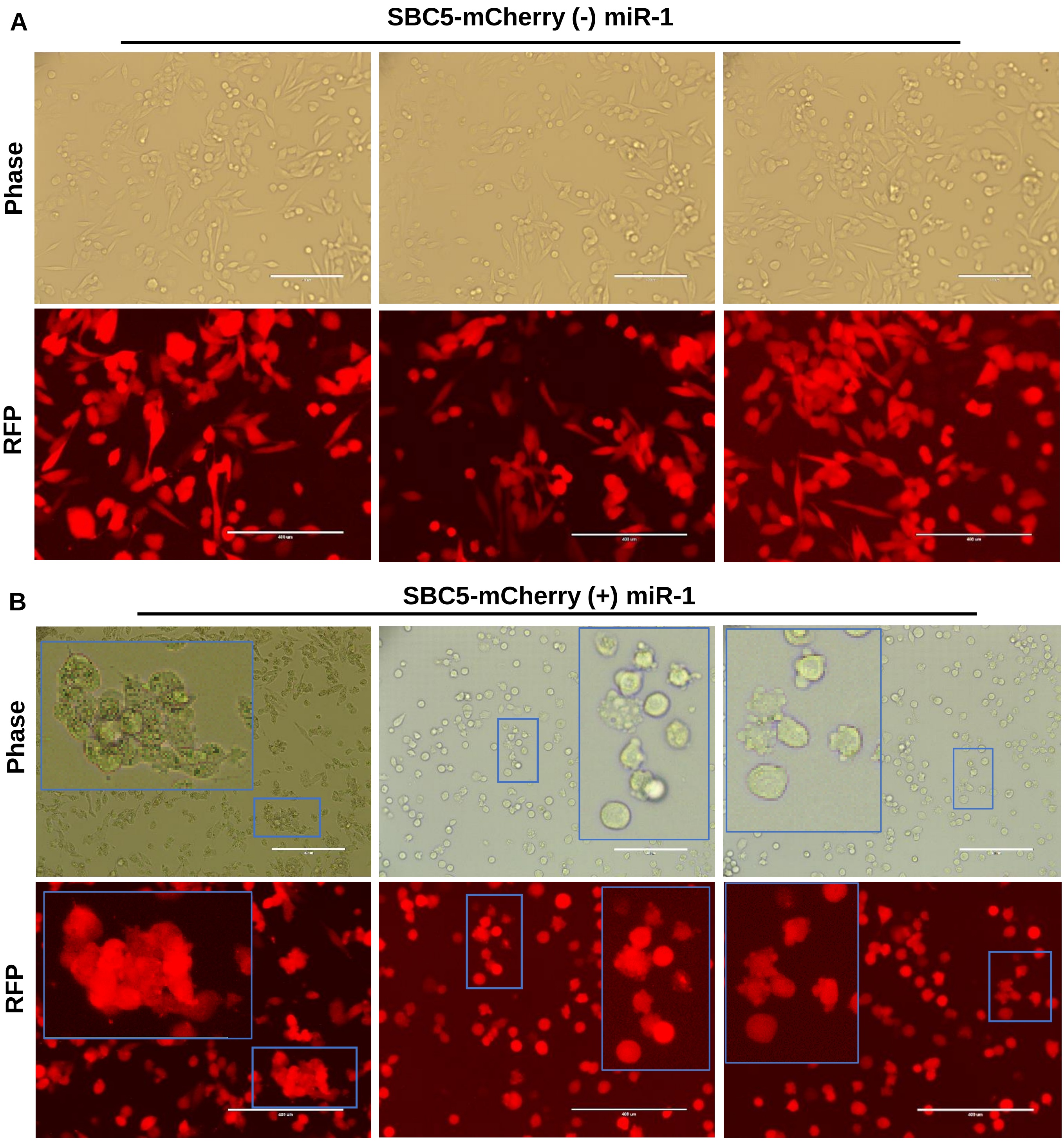

Figure S6: Exogenous expression of miR-1 induces apoptosis-like morphological changes in SBC5 cells. SBC5(-DOX-off)/SBC5-DOX-On-miR-1 cells were seeded in a 6-well plate, and DOX-On-miR-1 cells were induced with doxycycline (5µg/ml) and images were captured in phase contrast and RFP filters (as transduced cells express mCherry). **(A)** images of SBC5(-DOX-off) **(B)** images of SBC5-DOX-On-miR-1 cells. DOX-On-miR-1 cells show apoptosis-like morphologies in cells (RFP: red fluorescence protein).

Supplementary Figure S7

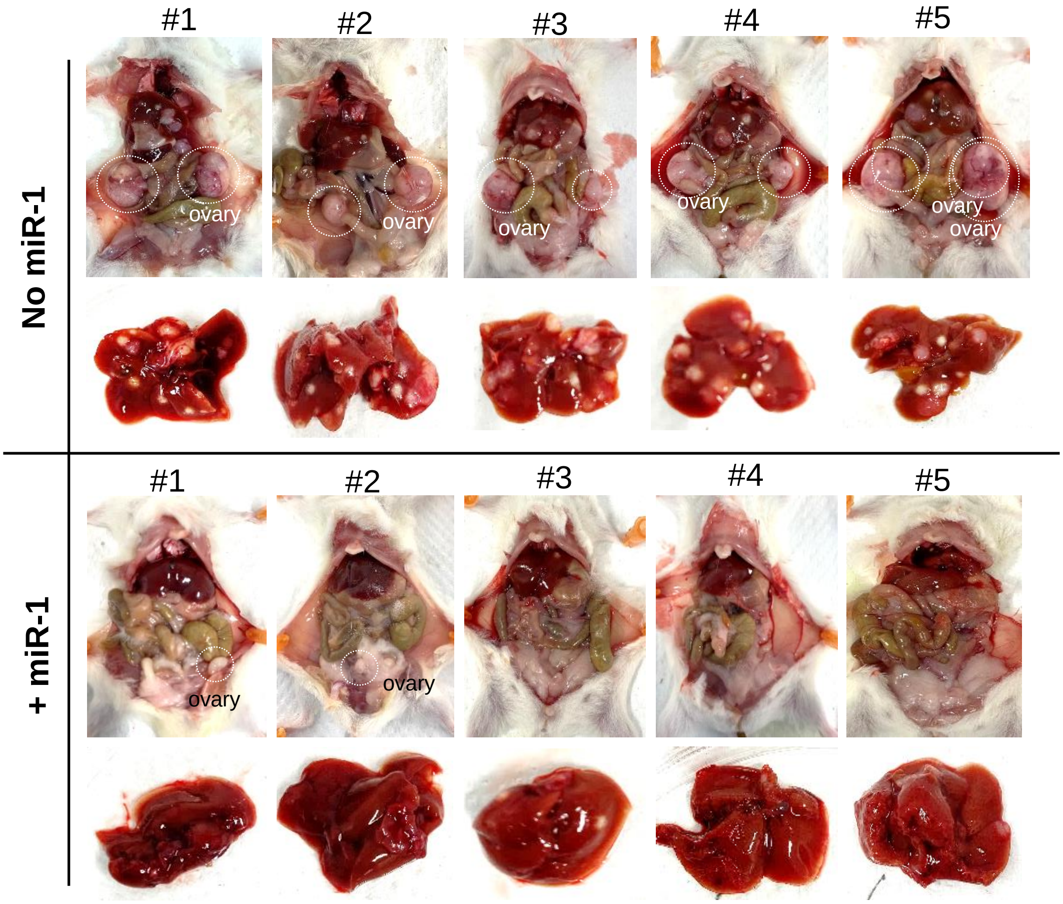

Figure S7: Ex-vivo digital images of euthanized dissected mice (intracardially injected with SBC5-DOX-off/SBC5-DOX-On-miR-1) showing metastasis in abdominal cavity organs such as liver and ovary (highlighted in white dotted circles) of mouse intracardially injected with SBC5 cells without miR-1 (upper panel) and with miR-1 (lower panel).

Supplementary Figure S8

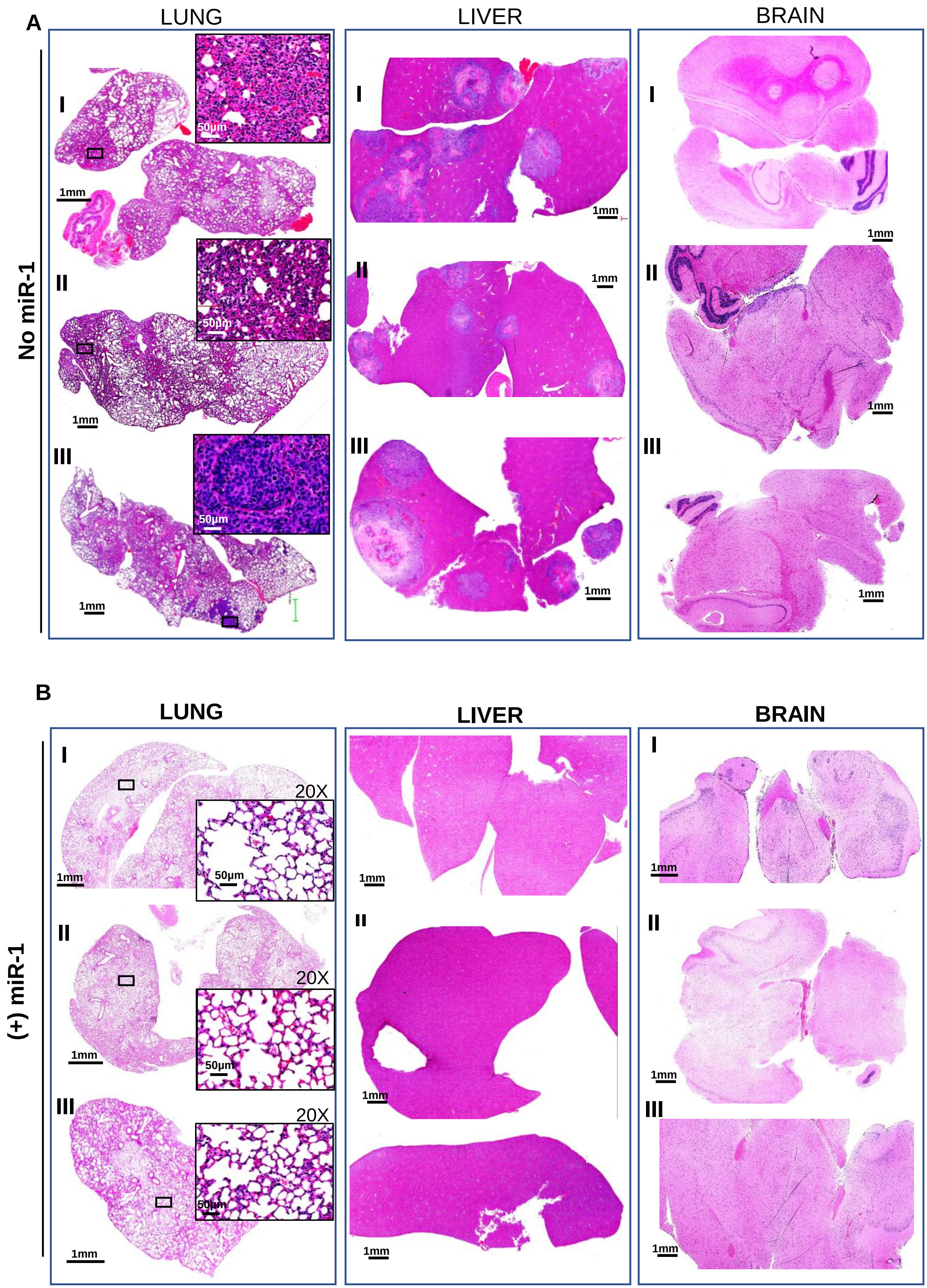

Figure S8: Tissue histology of lung, liver, and brain tissues using H&E staining, from the NSG mice intracardially injected with SBC5 cells (SBC5-DOX-off/SBC5-DOX-On-miR-1), **(A)** without miR-1 **(B)** with miR-1.

Supplementary Figure S9

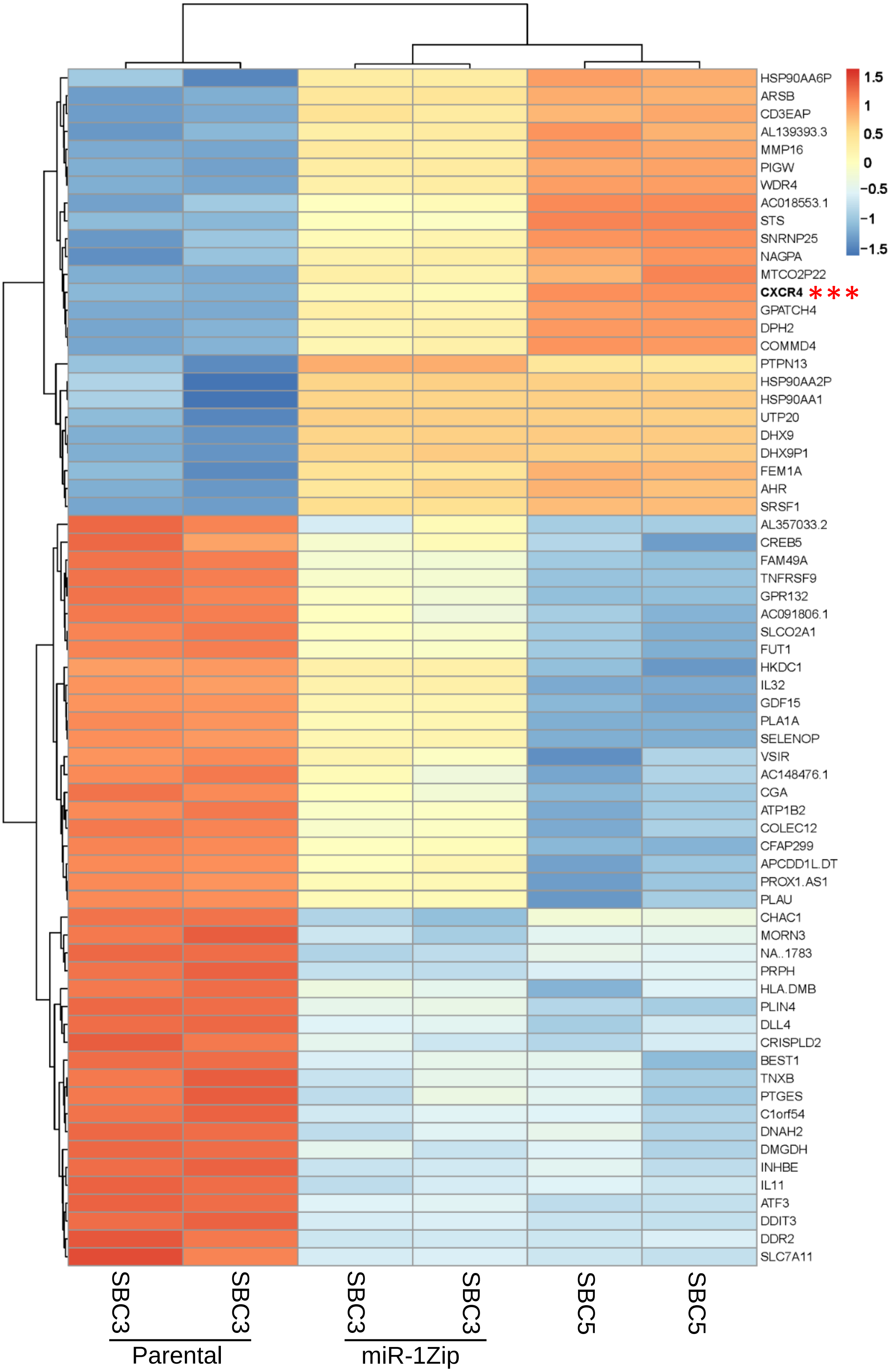

Figure S9: The clustered heatmap of top differentially expressed genes in SBC3, SBC3-miR-1Zip, and SBC5 cell lines. The blue to red transition indicate upregulation of the respective gene.

Supplementary Figure S10

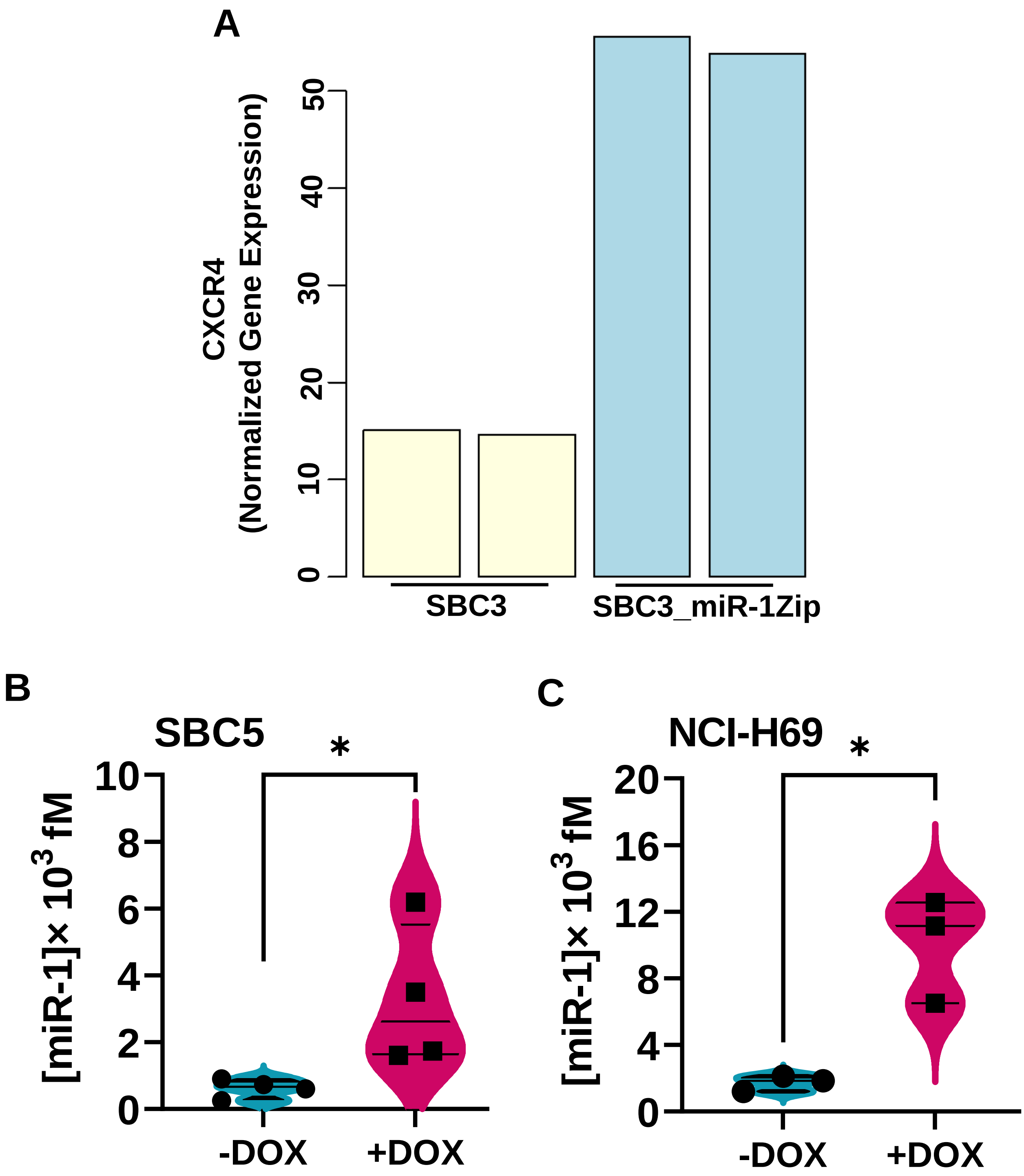

Figure S10: (A) Normalized expression of CXCR4 in the parental and miR-1Zip SBC3 cell line. SCLC patient dataset. **(B-C)** Violin with point graph representing the quantitative expression of miR-1 in –DOX and +DOX SBC5 and NCI-H69 cell lines. p-values are the results of the t-test for unpaired samples, $*p<0.05$.

Supplementary Figure S11

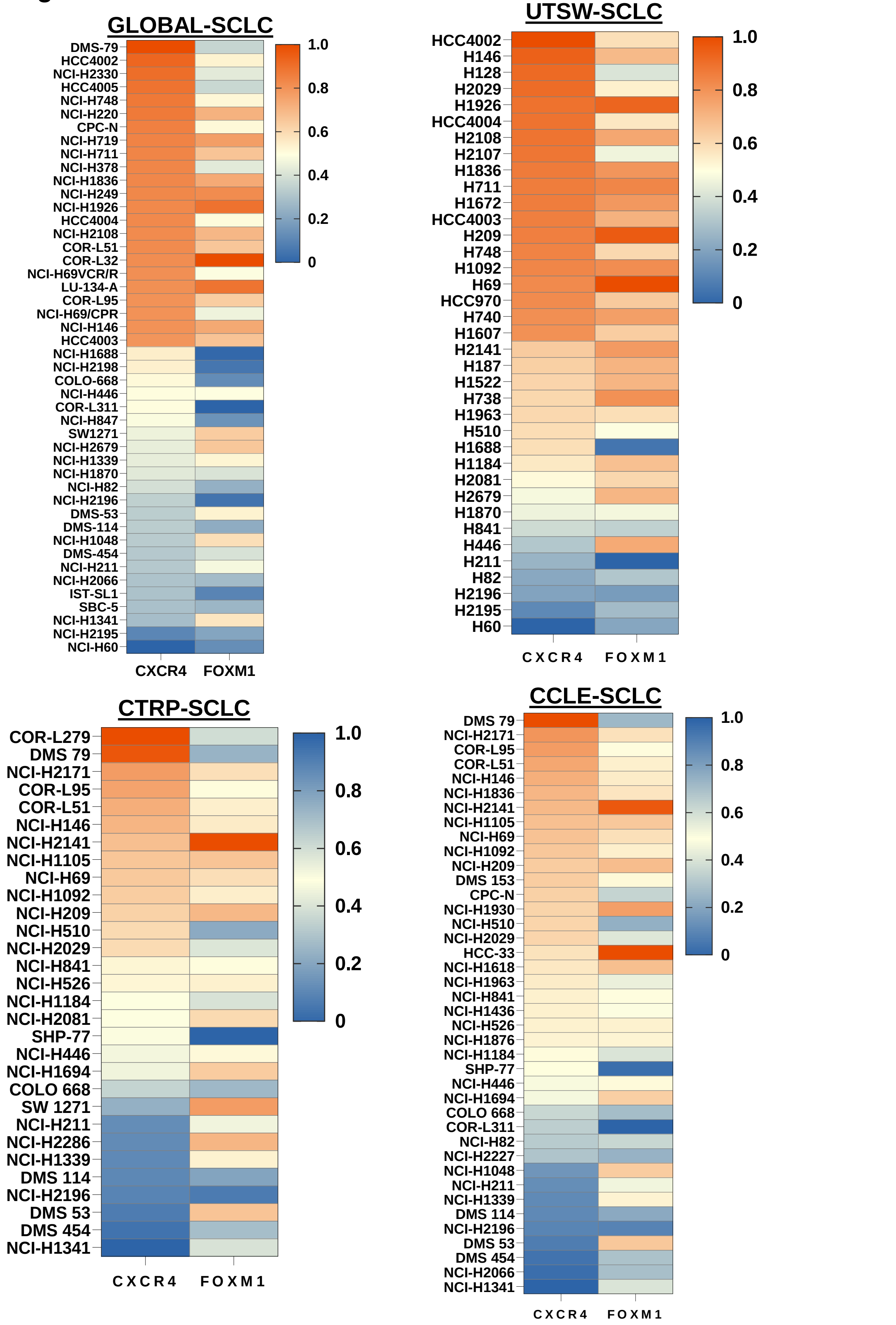

Figure S11: Heatmap for normalized expression of CXCR4 and FOXM1 in a panel of SCLC cell lines data sets from SclcCellMiner. (https://discover.nci.nih.gov/rsconnect/SclcCellMinerCDB/).

Supplementary Figure S12

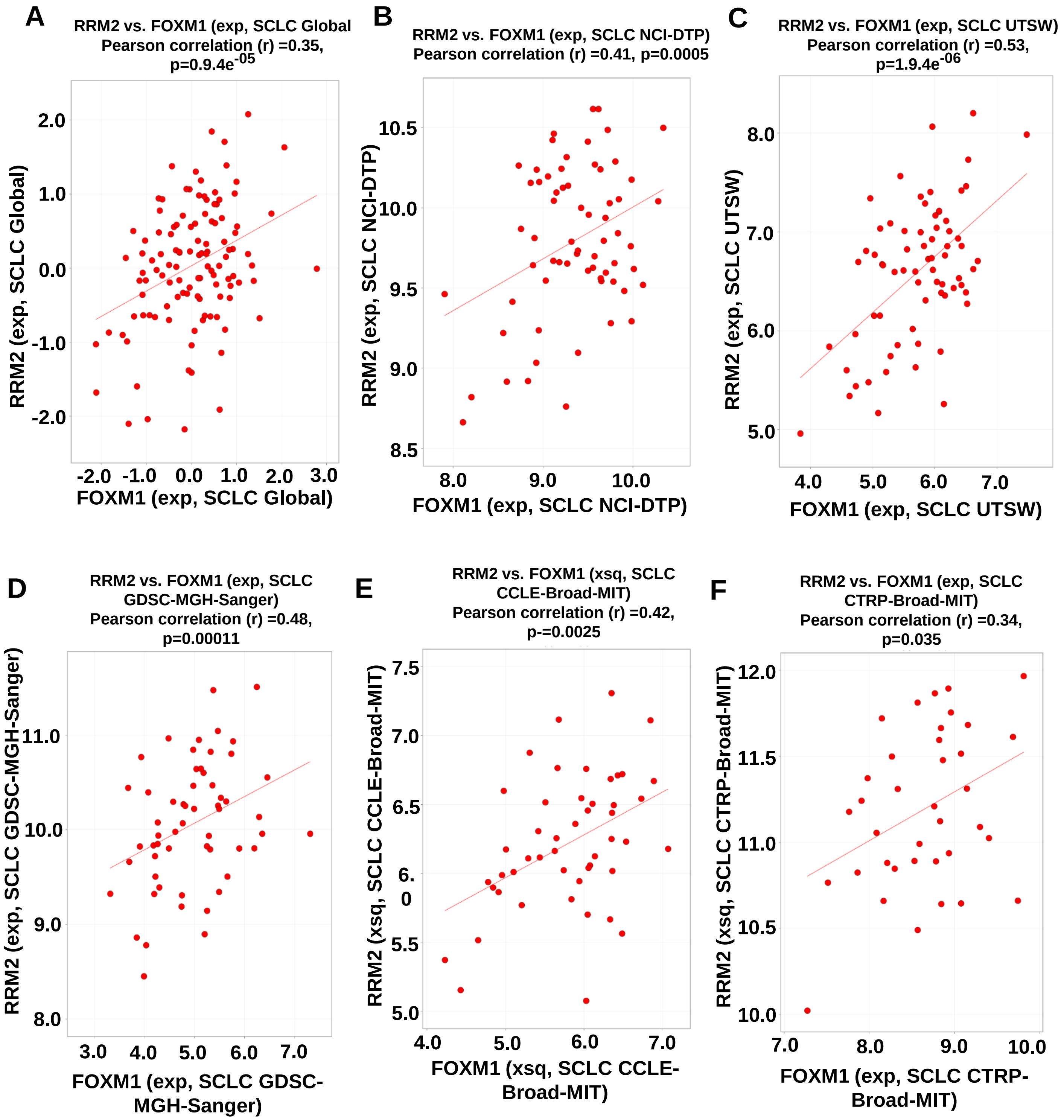

Figure S12: Transcriptomic correlation of RRM2 and FOXM1 in publically available data sets (https://discover.nci.nih.gov/rsconnect/SclcCellMinerCDB/) of SCLC. **(A-F)** RRM2 and FOXM1 show a positive correlation in the studied datasets.

Supplementary Figure S13

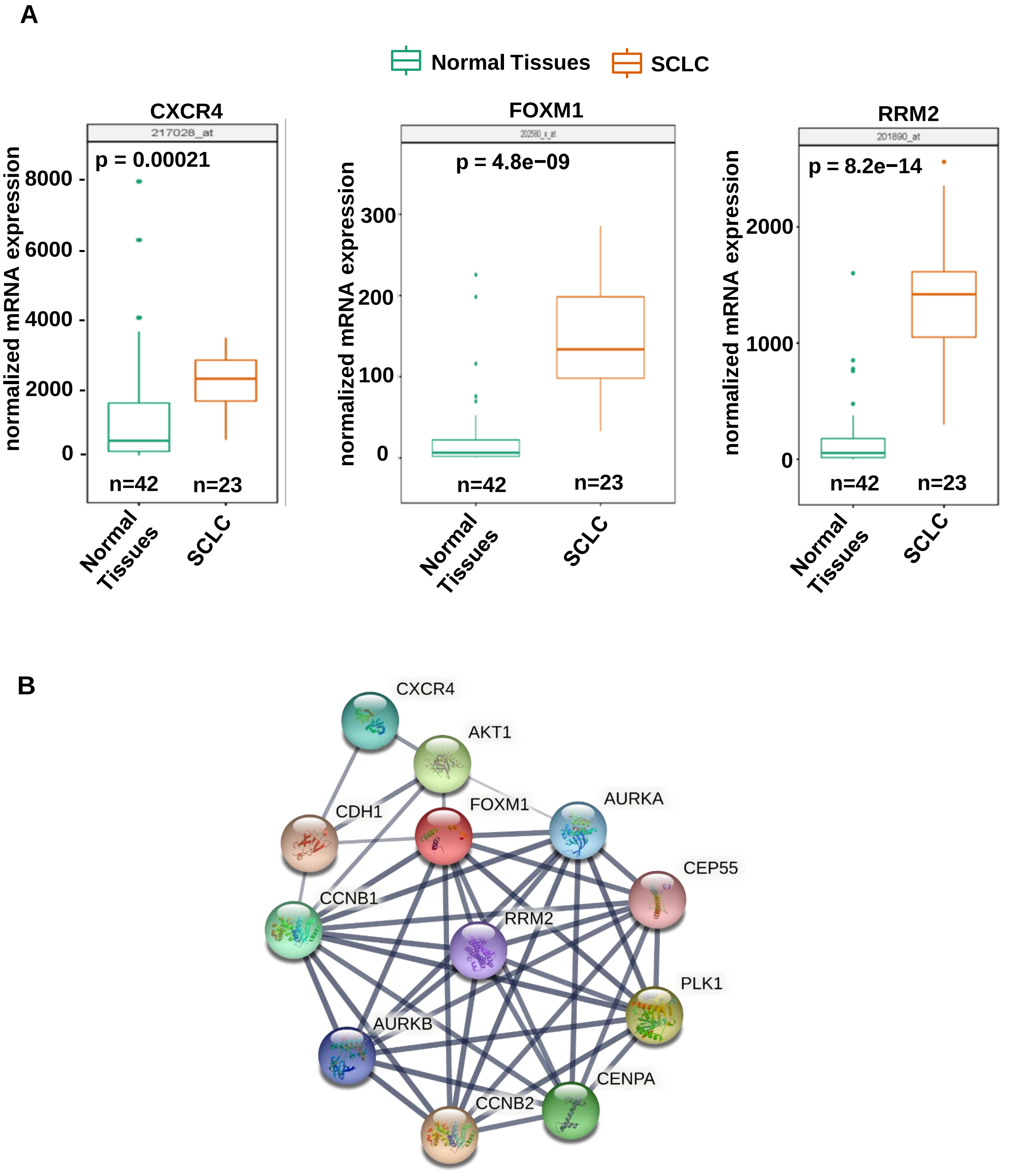

Figure S13. (A) Normalized expression of CXCR4, FOXM1, and RRM2 in SCLC patient dataset. **(B)** Interactome analysis of top differentially regulated genes in SBC5(-DOX-off)/SBC5-DOX-On-miR-1 cells**. (B).**

Supplementary Figure S14

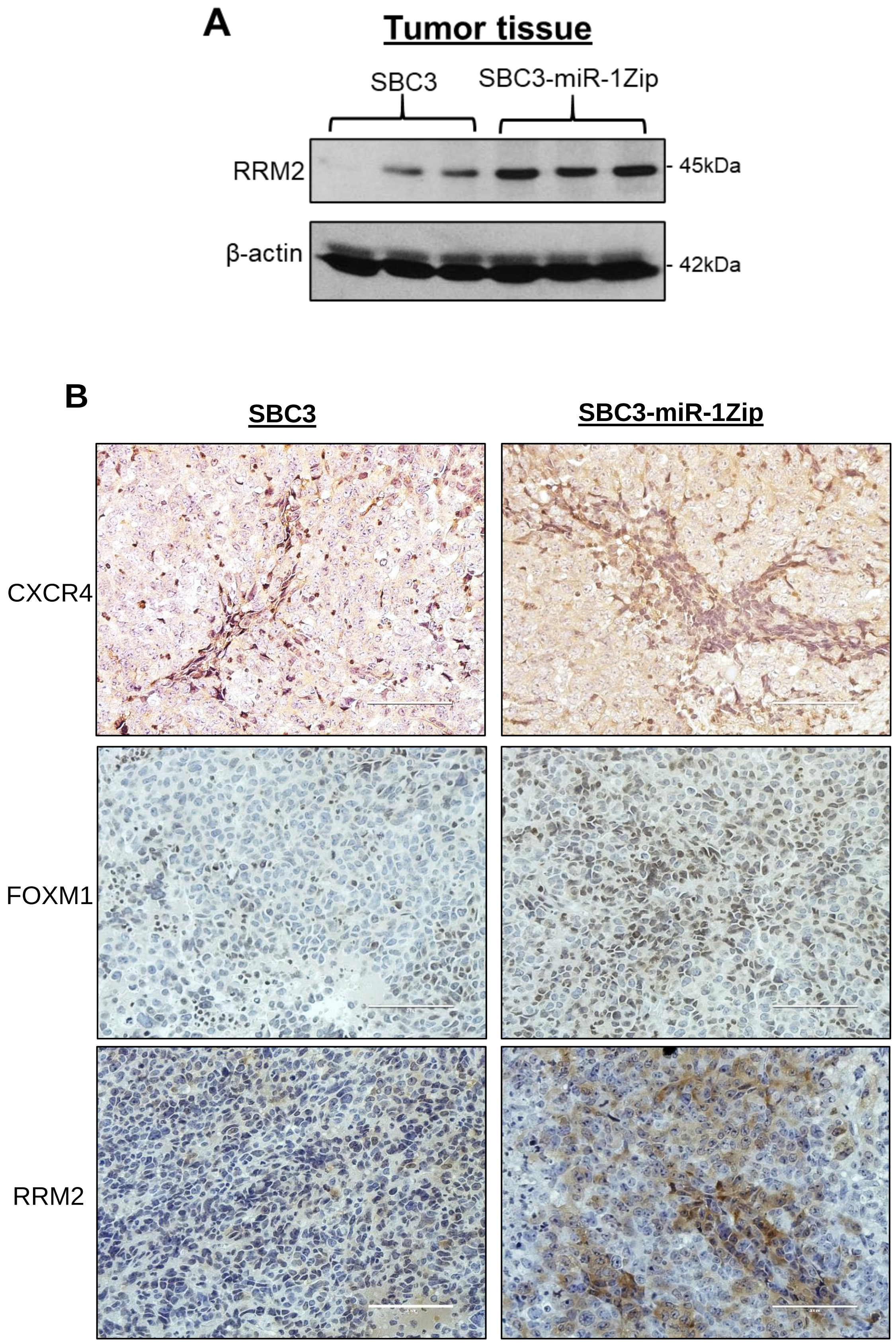

Figure S14: (A) RRM2 expression in the lysates of SBC3 and SBC3-miR-1Zip xenograft tumor tissues. **(B)** Immunohistochemistry analysis of xenograft tumor tissues of SBC3 and SBC3-miR-1Zip cells demonstrating the expression of CXCR4, FOXM1, and RRM2. The scale bar represents 200 µm.

Supplementary Figure S15

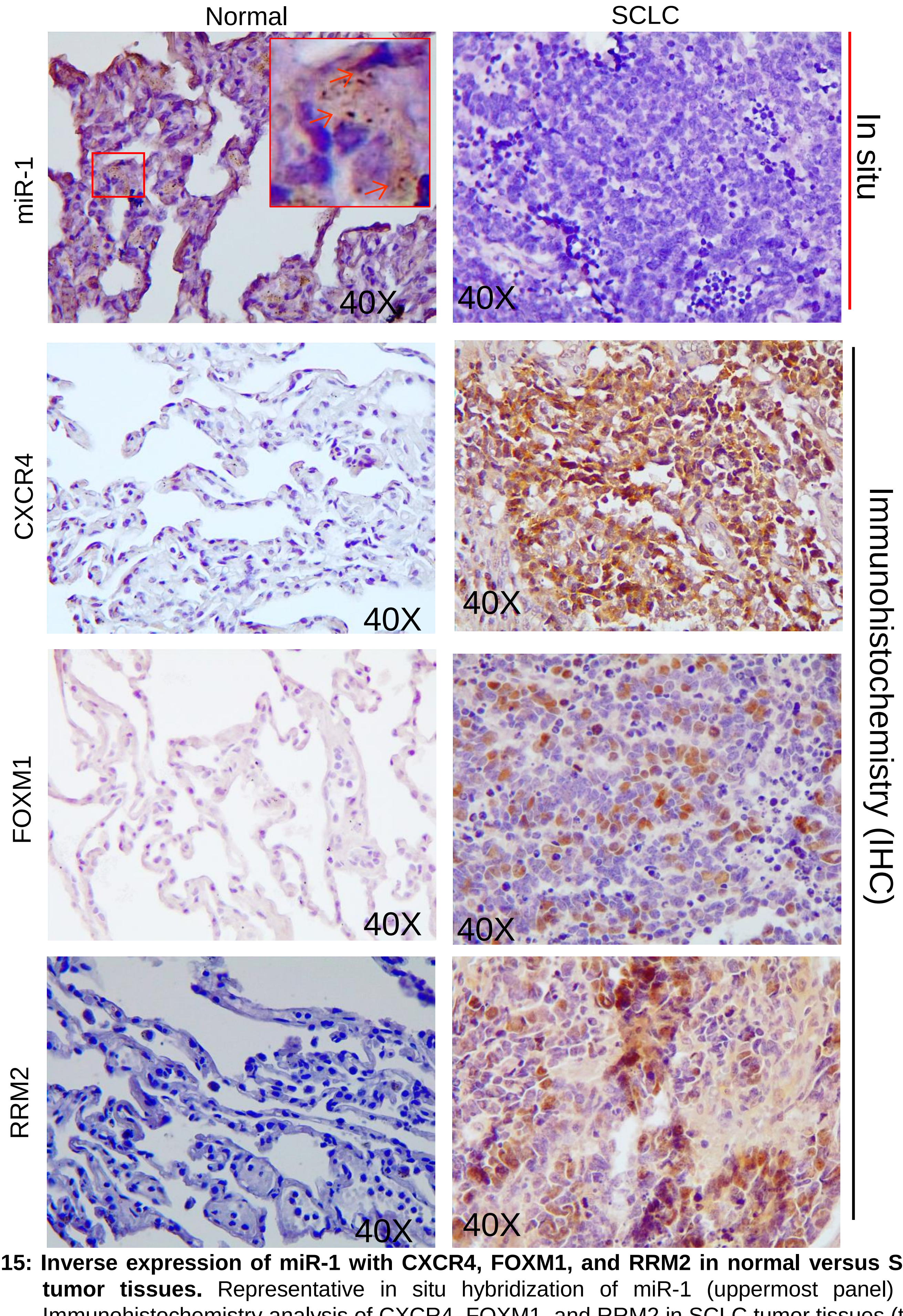

Figure S15: Inverse expression of miR-1 with CXCR4, FOXM1, and RRM2 in normal versus SCLC tumor tissues. Representative in situ hybridization of miR-1 (uppermost panel) **and** Immunohistochemistry analysis of CXCR4, FOXM1, and RRM2 in SCLC tumor tissues (three lower panels) using SCLC tissue microarray (BS04116a, US Biomax, Inc.).

Supplementary Figure S16

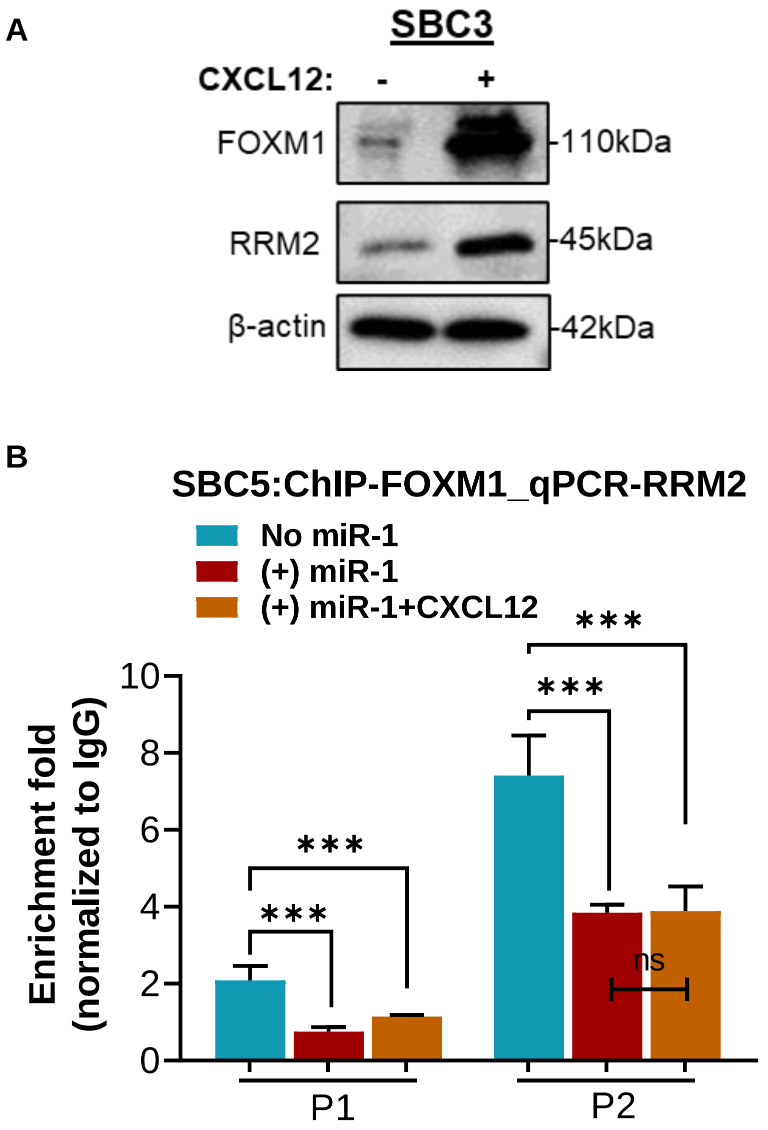

Figure S16: (A) FOXM1 and RRM2 expression in SBC3 cells treated with CXCL12 (100ng/ml, for 48-72h) through Western blot. **(B)** Chromatin immunoprecipitation of FOXM1 and RRM2 qPCR confirming the binding of FOXM1 with RRM2 promoter, miR-1 decreased the interaction of FOXM1 with RRM2 promoter, and the CXCL12 treatment (100ng/ml, for 48-72h) in the presence of miR-1 showed a non-significant change in the accessibility of RRM2 promoter to FOXM1 compared to +miR-1 group. Statistical significance was considered using ordinary one-way ANOVA. ***, $p<0.001$; ns, non-significant.

Supplementary Figure S17

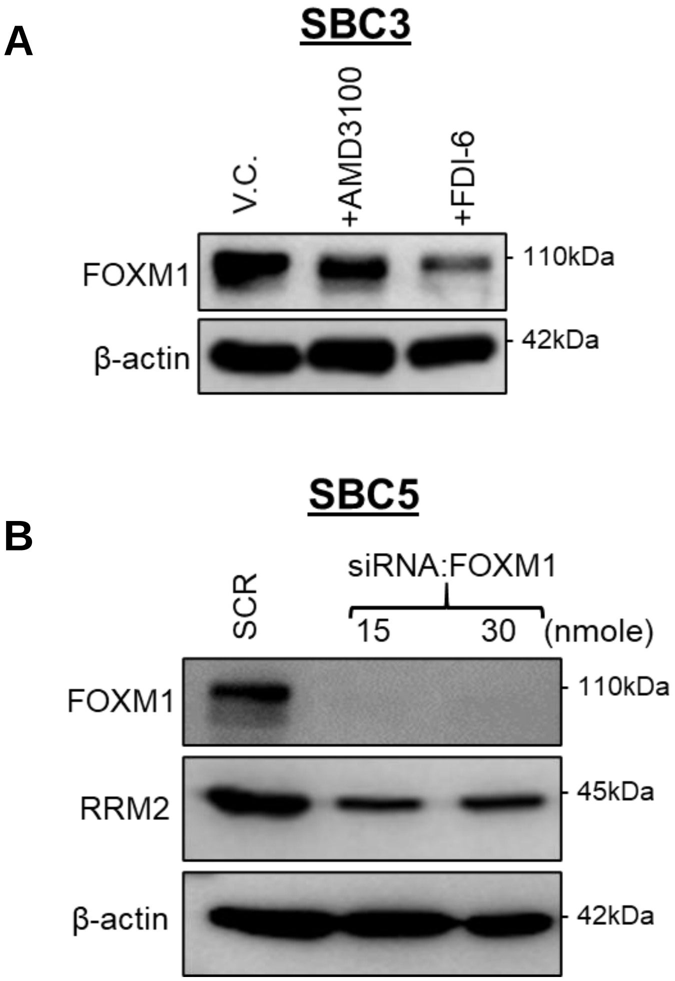

Figure S17: (A). FOXM1 expression in AMD3100 (CXCR4 inhibitor) and FDI-6 (FOXM1 inhibitor) treated SBC3 cells. SBC3 cells were treated with either AMD3100 or FDI-6, and the cells were harvested following 48 hrs of treatments and analyzed for FOXM1 expression through Western blot. **(B)** FOXM1 and RRM2 expression in SBC5 cells treated with FOXM1 siRNA. The cells were transfected with 15 and 30 nmole FOXM1 siRNA. Transfected cells were harvested following 48 hrs of transfection and analyzed for FOXM1 expression through Western blot..
