# Supplementary material for: MicroRNA-1 attenuates the growth and metastasis of small cell lung cancer through CXCR4/FOXM1/RRM2 axis
Source: Mol Cancer. 2023 Jan 4;22:1. doi: 10.1186/s12943-022-01695-6 (PMC9811802; doi:10.1186/s12943-022-01695-6)
Supplement: Supplementary file 2 — Additional file 2. [file 12943_2022_1695_MOESM2_ESM.docx]

**MicroRNA-1 attenuates the growth and metastasis of small cell lung cancer through CXCR4/FOXM1/RRM2 axis**

Parvez Khan^a,^ Jawed Akhtar Siddiqui^a^, Prakash G. Kshirsagar^a^, Ramakanth Chirravuri Venkata^a^, Shailendra Kumar Maurya^a^, Tamara Mirzapoiazova^b^, Naveenkumar Perumal^a^, Sanjib Chaudhary^a^, Ranjana Kumari Kanchan^a^, Mahek Fatima^a^, Md Arafat Khan^a^, Asad Ur Rehman^a^, Imayavaramban Lakshmanan^a,^ Sidharth Mahapatra^a,c,d^, Geoffrey A. Talmon^e^, Prakash Kulkarni^b^, Apar K Ganti^f^, Maneesh Jain^a,c^, Ravi Salgia^b^, Surinder Kumar Batra^a,c,g^, Mohd Wasim Nasser^a,c,*^

*^a^Department of Biochemistry and Molecular Biology,* *University of Nebraska Medical Center, Omaha, NE-68198, USA.*

*^b^Department of Medical Oncology and Therapeutics Research, City of Hope National Medical Center and Beckman Research Institute, Duarte-91010, CA, USA*

*^c^Fred and Pamela Buffett Cancer Center, ^d^Department of Pediatrics, University of Nebraska Medical Center, Omaha, NE-68198, USA.*

*^e^Department of Pathology and Microbiology, University of Nebraska Medical Center, Omaha, NE, USA*

*^f^Division of Oncology-Hematology, Department of Internal Medicine, VA-Nebraska Western Iowa Health Care System, Omaha, NE, 68105, USA;* *Division of Oncology-Hematology, Department of Internal Medicine, University of Nebraska Medical Center, Omaha, NE, 68198, USA.*

*^g^Eppley Institute for Research in Cancer and Allied Diseases, University of Nebraska Medical Center, Omaha, NE-68198, USA.*

*Running title: role of miR-1 in SCLC.*

***To whom all correspondence should be addressed:**

Mohd Wasim Nasser, Ph.D.

Associate Professor

Department of Biochemistry and Molecular Biology

University of Nebraska Medical Center,

Omaha, NE-68198, USA.

*E-mail:* [*wasim.nasser@unmc.edu*](mailto:wasim.nasser@unmc.edu)

Phone: +1- 402-5592027

| **Supplementary method/data/Table** | **Page** |
| --- | --- |
| 1. Materials | 3 |
| 2. Synthesis of DNA-AuNPs | 3 |
| 3. Physicochemical characterization of the DNA-AuNPs | 4 |
| 4. In-situ hybridization | 4-5 |
| 5. TaqMan assay for miR-1 expression | 5 |
| 6. 3D spheroid assay | 5-6 |
| 7. Colony formation assay | 6 |
| 8. Real-time scratch wound healing assay | 6-7 |
| 9. Chromatin immunoprecipitation and qPCR | 7-9 |
| 10. CXCR4 3'-UTR dual luciferase assay | 9-11 |
| 11. Flow cytometry | 11-12 |
| 12. Immunoblot analysis | 12 |
| 13. Immunohistochemistry | 13 |
| 14. *Table-1* Size, surface charge, and TEM characterization of AuNP | 14 |
| 15. *Table S2*. Summary of spectroscopic characterization of AuNPs (UV-Vis, DLS, NTA, TEM) | 15 |
| 16. *Table S3*. Quantification of the attached DNA-probe per gold nanoparticle (AuNP) | 16 |
| 17. *Table S4.* Summary of proposed optimal conditions tested in miRNA assay | 17 |
| 18. *Table S5.* Details of antibodies and reagents used in the study. | 18 |

**Material and Methods**

**Materials**

All other chemical reagents for DNA-AuNPs synthesis were obtained from Millipore-Sigma or Fisher Scientific and used without any purification unless otherwise mentioned. Acid and amine derivatives of Alkyl-PEG600 thiols were supplied by Advanced BioChemicals, LLC (Georgia, USA). The SM(PEG)2 heterobifunctional linker was purchased from Pierce™ (Illinois, USA). The total RNA isolation kit mirVana™ (Cat. No. AM1560,) and MagMAX™ mirVana™ (Cat. No. A27828) were bought from Applied Biosystems. The synthetic oligonucleotides (Table S1) were purchased from Integrated DNA Technologies (Iowa, USA).

**Synthesis of DNA-AuNPs**

All glassware used in gold nanoprobe synthesis and PTFE magnetic stir bars, were washed with Aqua-regia (HCl (37%) and HNO3 (65%) in a 3:1 volumetric ratio), followed by ultrapure deionized water. First, the gold nanoparticle seeds (size ~13 nm) were prepared using the Grabar synthesis protocol and grown to ~35 nm particle size using the seed growth method. Next, the PEGylation of the AuNPs was performed by following standard protocol from the previous publication [1-3]. Briefly, we added 2-4 nM of 35 nm AuNP to the 20:80 mixture of amino- and carboxy-terminated PEG thiols in 95% ethanol (𝑥NH2 = 0.15), and 50 mM NaHCO3 (pH 8.5). The reaction mixture was stirred for the next 72 h at RT, and the PEG-coated AuNPs were purified and washed by ultrafiltration method using 50 mM NaHCO3 buffer. Next, in the two separate reaction sets, the TCEP-deprotected DNA-probes that are specific to miRNA-1 (3P) and miRNA-1 (5P) were coupled with PEG-passivated AuNPs, using an SM (PEG)2 linker molecule [1]. Briefly, Linker-AuNPs (5-8 nM), phosphate buffer (25 mM, pH 7.0), and NaCl (200 mM) were mixed with freshly deprotected DNA-probes (~15 µM). The reaction mixture was shaken overnight at 4°C on the thermomixture. Followed by the mixture was centrifuged (10,000 rpm, 10 min, 4°C), washed three times with ice-cold 50 mM NaHCO3, and stored at 4°C.

**Physicochemical characterization of the DNA-AuNPs**

Upon synthesis of DNA-AuNPs, the UV visible, DLS, NTA, and zeta (ζ) potential analyses were performed on both the bare and DNA-probe-modified AuNPs. The transmission electron microscopy (TEM) imaging was done on the fresh aliquots of AuNPs or DNA-AuNPs by adding a few drops of the AuNPs onto the carbon-coated copper grid (200 mesh, Ted Pella, Inc. USA). All particles were imaged with the FEI Tecnai G2 Spirit transmission electron microscope. The average size of AuNPs was determined via processing TEM images with ImageJ software (https://imagej.nih.gov/ij/). We performed Agarose gel electrophoresis (for 40 min, at 100 V) to assess the formation of DNA-AuNP conjugates by analyzing electrophoretic mobility of the DNA-AuNP and Linker-AuNPs. We recorded the gel image with a digital camera and processed the picture only with small linear contrast. Finally, we used fluorescence and ICP-base analysis to determine the number of surface oligos per AuNP (NOligo). All the nanoparticle characterized data, and quantification data for the NOligos [1, 2] are summarized in **Table S2** and **Table S3**, respectively.

**In-situ hybridization**

The expression level of miR-1 was determined employing *in situ* hybridization (ISH). For this, we used a commercially available, single-color human or mouse-specific 20-paired double-Z oligonucleotide probe (Advanced Cell Diagnostics) as described previously [4]. Briefly, tissue sections from the SCLC patient tissue microarrays (Cat#BS04116a, U.S. Biomax, Inc.) were used for ISH. For de-paraffinization, the tissue slides were baked at 60 °C for 1 h, followed by sections were incubated with protease for 30 minutes at 40 °C. The pre-heated probes of miR-1 were hybridized for 2 hours at 40 °C. Following the manufacturer's protocol, signal amplification and washing steps were performed using the HybEZ Hybridization System following the hybridization step. The signals from hybridized probes were detected using sequential chromogenic reactions of brown chromogens. For the detection of miR-1 transcripts, we used a commercially available kit (ISH with RNAscope® 2.5 HD Assay- BROWN, RNAscope®)78. The images were captured using a 40x and 100x Bright-field objective.

**TaqMan assay for miR-1 expression**

Total RNA was isolated using mirVana™ miRNA isolation kit, Catalogue number: AM1561 (Invitrogen™, Thermo Scientific, USA). Stem-loop primers specific for miR-1-3p (Cat#4427975, Applied Biosystem) were used for the reverse transcription reaction with RNU6B (Cat#4427975, Applied Biosystem) as an internal control using microRNA Reverse Transcription Kit (Applied Biosystem). A single-step real-time PCR system (Applied Biosystem) was used to perform miR-1 specific TaqMan assay. The expression level of miR-1 was normalized to RNU6B and quantified using ∆∆Ct method.

**3D spheroid assay**

SBC3, SBC3-miR-1Zip, and SBC5-DOX-On miR-1 cells (stably transduced with NucLight Red or Green Lentivirus particles, Essen BioScience) were plated at a density of 9000-10000 cells/well in an ultra-low attachment 96-well plate and allowed to form spheroid (~72 h). Spheroid growth was monitored using red/green fluorescence as an indicator of cell growth/viability as described previously [5]. In the case of SBC5-DOX-On miR-1 cells, the expression of miR-1 was induced using doxycycline (5µg/ml). To monitor spheroid growth over time, the plate was placed in the IncuCyte S3 Live Cell Imaging System (Essen BioScience), and images were acquired every 4 h for 6 days. Data analysis was performed using IncuCyte ZOOM software using a red/green fluorescence mask indicating spheroid viability.

**Colony formation assay**

Nearly, 500-1000 cells (SBC3/SBC3-miR-1Zip, SBC5 (-DOX-off) Vs. SBC5-DOX-On-miR-1) were seeded in a 6-well plate with the complete medium. In case of SBC3/SBC3-miR-1Zip cells, the cells were allowed to grow in complete medium (with or without CXCL12, a ligand for CXCR4) for 9-10 days to allow colony formation. On the other hand, in case of SBC5 (DOX inducible miR-1 overexpression clones), the cells were allowed to grow for 2-3 days, and expression of miR-1 was induced using 5µg/ml doxycycline and plates were incubated for 9-10 days. The final colonies were fixed and stained using 0.25% crystal violet (dissolved in 30% methanol). The number of colonies was counted and quantified using the Image-J image processing tool (https://imagej.nih.gov/ij/).

**Real-time scratch wound healing assay**

SBC3/SBC3-miR-1Zip, SBC5 (-DOX-off), SBC5-DOX-On-miR-1 cells were seeded in a 96-well ImageLock (Essen BioScience, Ann Arbor, MI, USA) plate to reach 90% confluence on the next day. Following cell adherence and growth on the next day, simultaneously in each well of 96-well plate, uniform wounds were created using the WoundMaker (Essen BioScience) tool. Cells were washed with serum-free medium to remove floaters or free-floating cells and replenished with the complete medium. To monitor wound closure or healing, the plates were placed in the IncuCyte S3 Live Cell Imaging System (Essen BioScience), and images were captured every hour up to 72-80 h. Data analysis was performed using IncuCyte software (using a set confluence mask) to measure relative wound density over time.

**Chromatin immunoprecipitation and qPCR**

Chromatin immunoprecipitation (ChIP) was performed with minor modifications as described previously [6]. Briefly, 15 x 10^6^ cells were cross-linked with 1% formaldehyde for 10 min at room temperature. The cross-linking was stopped by adding 0.125 M glycine for 5 min, and cells were washed with chilled PBS (2X), scraped, and collected by centrifugation (820 x g, 5 min, 4°C). The cell pellets were lysed in 400 μl of SDS lysis buffer (1% SDS, 10 mM EDTA, 50 mM Tris-HCl (pH 8.0), 1X protease inhibitor cocktail) and incubated for 15-20 min on ice. The cell extracts were sonicated (Branson sonifier) at 25% amplitude with 3-4 pulse (20 s ON and 30 s OFF pulse on ice) to generate ∼500 nucleotide chromatin fragments and centrifuged at 17,000 x g for 10 min at 4°C. Nearly, 10% of the supernatant was saved as input DNA and stored at -80°C before downstream processing. The remaining chromatin samples were precleared with 50 μl of protein A/G PLUS-Agarose beads (Santa Cruz Biotechnology) for 1-2 h with rotation at 4°C, centrifuged (600 x g, 5 min), and diluted 10-fold in a ChIP dilution buffer (0.01% SDS, 1.1% Triton X-100, 1.2 mM EDTA, 16.7 mM Tris-HC (pH 8.0), 167 mM NaCl, 1X protease inhibitor). The chromatin fraction was then equally divided and immunoprecipitated with either FOXM1 (Cat#20459, Cell Signaling Technology, 1:100 dilution) or rabbit IgG along with 60 μl of protein A/G PLUS-agarose beads for overnight rocking in 4°C. Next day, the beads were washed with the following buffers- low salt wash buffer (0.1% SDS, 1% Triton X-100, 2 mM EDTA, 20 mM Tris-HCl, pH 8.1, 150 mM NaCl, 1X protease inhibitor), high salt immune complex wash buffer (0.1% SDS, 1% Triton X-100, 2 mM EDTA, 20 mM Tris-HCl, pH 8.1, 500 mM NaCl, 1X protease inhibitor), LiCl immune complex wash buffer (0.25 M LiCl, 1% NP-40, 1% deoxycholic acid, 1 mM EDTA, 10 mM Tris-HCl (pH 8.1), 1X protease inhibitor), and TE (10 mM Tris-HCl, 1 mM EDTA, 1X protease inhibitor). The immune complexes were eluted in SDS elution buffer (1% SDS and 50 mM NaHCO_3_), reversed cross-linked (5M NaCl) at 65°C for 6h, digested with proteinase K at 45 °C for 1h, and the DNA was extracted using the ChIP DNA Clean and concentrator (Zymo research). The enriched ChIP DNA was analyzed by quantitative real-time PCR using Syber green on the CFX Connect Real-Time PCR Detection System (Bio-Rad Laboratories, Hercules, CA). The primers used in the study are-P1 F/R, P2 F/R, P3 F/R, and P4 F/R **(details provided below)**. The coimmunoprecipitated gene fragments were represented as a fold enrichment method based on Ct values (2^-(Ct(FOXM1 IP) – Ct(IgG))^).

**FOXM1-RRM2 Chip Assay:**

***RRM2 promoter***

**Homo sapiens chromosome 2, GRCh38.p13 Primary Assembly**

NCBI Reference Sequence: NC_000002.12

FoxM1 binding seq: (C/TAAACA)

ORIGIN

1 ggctagcagc agcctgcggc gggcgctctc ccgggagtgg ctgcaccgcc cgacctcccc

61 ggaggcggaa ccgcccgcat tgccgcgtgg ccctgggcgc cgccacctcc tccgcagcgg

121 ggcaaagttg ccggacctgg gggcaggagg gccacgccga gatgactcag gtttagcgcg

181 ggaggggagg atggcgactt cacccggcct ttaacaacac gtacgcatct ttcggcgtct

241 tctacaatgg ctatgttaat tacgtggcca ggaactaaac tatcaatgaa gccacctctg

301 actacttcag ttacagtgag tttaacagga gcaaaaaagc acgtggcgcc ctagggcaac

361 cgaaacgagg gttttagacg ctgattatgg gaaattgaaa tctgagttga gtatgagatg

421 acaccaataa attataattt tgttagataa tagctttatc agccataaag taatcaataa

**P1-F**

481 aaataccagt ttcctggaga tggatgcttt agtgtgtttg gggtgaaaa***t ggcgatgaat***

541 ***ggcgagttg***c tt**taaaca**aa tcatggtaca ccaaagtttt agttgtggct ttgtgtaagg

601 aatgtgatgg gcacttattc ctgcaacacg agaatactat gatttacaag tccgtagtac

**P1-R**

661 ttttaagaaa tgaga***gaaac agacctaggt gggga***gggta cctgtcccac cccaccctct

721 ttaaagtatc ttatctagaa aaggctttgt gaaaaaaaaa gtcccgggtc tctctcaata

781 acagccctga gcgcagctgt tgaagctttc tcaggttaat gatttctttc ttggatctta

841 aagtttcttt ctcttccttt atttttggca ttttgcccgt tgcagggcct ggcaaatcag

901 aaagccacat agaaaattaa atgaaagcta ttgctaagtt ccagtctcta caccagtgga

961 gttttcaaac tcctcttcag catatttgac gcccaatgag tagtacatta attcctagtc

1021 ctaaaatcat tctgtgaact ttctcccagg aatttttgct cagtttgcaa ttaaaacaac

**P2-F**

1081 tttttttctt ctctt***tttaa tggcagaggc ggggt***ctcac tatgttgccc aggatggtct

1141 ccagctcctg gcct**caaaca** atcctcctgc cttggcctcc caaagtgctg ggattacagg

**P2-R**

1201 cgtgagccac cgcgcccagc cacagtttgc aattcttaag ***gcaagggtga caataggggt***

1261 caggggtctg acaggagaca ggatttctgt ggaaaactgc accaagggcc ttctcgccat

1321 gtcccgtagt ttgaaggttt acaaaggact gcacatttta catgagtcat ctcaacgaac

1381 gctctcctca ccgcattaac agtccacgcg gttacgagtc ccattttact cacggggaca

1441 ccgaatctgt aagaagcctg gtcgcttgtc ccagcaaaac gagccacggg gctcagcggc

1501 cctaactttt aggctgtagg gtcctcgccg accaccccgc caaaatgtca ggcctcgggg

1561 cccttgcacc cccaccgcag ggacacggat cgaaagggtc gcagcaacgc ctcccccgca

1621 cccaggagcg ttttccaggc ctttgcacca acctcgttgg ctaagccccc tgcccggcgg

**P3-F**

1681 cggcccggct gggaggaggt gctttcggga ggcggggccg c***ggcccgggg atcctctc***gc

**P3-R**

1741 gcccgcgggc tccaatcgct gctcctcacg c***aatcctaaa cggttcccgg g***cgaaccggg

1801 gcccgcgcgc gccaaggccg ccgagaccct caggggctgc ggccctggtc ccgcgggacc

1861 tgtgggggcc tgggcggcgg cgcccccgac ccagccagcg gacgggccgg ggggggaacc

1921 gggaggtccc ggggggcgtc cacgggggtg tccccggggg tctccggaag gcgccggcgg

1981 aggctcccgc gctgcgcttg aaaatcgcgc gcggccccgc ggccagcctg ggtaggggca

2041 aggcgcagcc a**atg**ggaagg gtcggaggca tggcacagcc aatgggaagg gccggggcac

2101 caaagccaat gggaagggcc gggagcgcgc ggcgcgggag atttaaaggc tgctggagtg

2161 aggggtcgcc cgtgcaccct gtcccagccg tcctgtcctg gctgctcgct ctgcttcgct

2221 gcgcctccac tatgctctcc ctccgtgtcc cgctcgcgcc catcacggac ccgcagcagc

2281 tgcagctctc gccgctgaag gggctcagct tggtcgacaa ggagaacacg gtgagcccgc

***Primer sequences for FOXM1-RRM2 Chip assay***

RM2P1-F: 5’-TGGCGATGAATGGCGAGTTG-3’

RM2P1-R: 5’-TCCCCACCTAGGTCTGTTTC-3’

RM2P2-F: 5’-GGCCCGGGGATCCTCTC-3’

RM2P2-R: 5’-CCCGGGAACCGTTTAGGATT-3’

RM2P3-F: 5’-TTTAATGGCAGAGGCGGGGT-3’

RM2P3-R: 5’-ACCCCTATTGTCACCCTTGC-3’

**Negative control (10 kb upstream)**

NC_Chip_RRM2 F: 5’-AGAGCTGGGAACCACTGACA-3’

NC_Chip_RRM2 R: 5’-GTTGCCCAGGCTGATCTTGA-3’

**miR-1:CXCR4 3'-UTR dual luciferase assay**

CXCR4 3'-UTR luciferase assay was performed as described previously [7]. Briefly, the primers for CXCR4-UTR, forward: 5'-CATATTGATGTGTGTCTAGGC-3' and reverse: 5'-CTGAAATCAACCCACTCCTG-3' were designed using the NCBI Primer blast tool (https:// www.ncbi.nlm.nih.gov/tools/ primer-blast/) and were procured from Euphorins (San Diego, CA, USA). Following PCR amplification of CXCR4 3' UTR, miR-1 binding/seed sequences (3' UTR Wild type) and mutated seed sequence (3' UTR Mutant type) were cloned into the pGL3 vector (Promega) expressing firefly luciferase. For the dual-luciferase assay, nearly 3X10^5^cells/well (SBC5) were seeded in a 12 well plate and then co-transfected with CXCR4 3'-UTR-wild-pGL3/3'-UTR-mutant-pGL3 plasmid, pRL-TK plasmid (Promega) expressing Renilla luciferase (internal control), miR-1 mimic and negative control. Following 48 h of transfection, luciferase activity was measured using the Dual-Luciferase Reporter Assay System (Promega, Madison, WI) using a Luminometer (Agilent, Biotek, CA, USA).

**MiR-1-3p CXCR4 3’UTR:**

CACAGATGTAAAAGACTTTTTTTTATACGATAAATAACTTTTTTTTAAGTTACACATTTTTCAGATATAAAAGACTGACCAATATTGTACAGTTTTTATTGCTTGTTGGATTTTTGTCTTGTGTTTCTTTAGTTTTTGTGAAGTTTAATTGACTTATTTATATAAATTTTTTTTGTTT**CATATTGATGTGTGTCTAGGC**AGGACCTGTGGCCAAGTTCTTAGTTGCTGTATGTCTCGTGGTAGGACTGTAGAAAAGGGAACTGAACATTCCAGAGCGTGTAGTGAATCACGTAAAGCTAGAAATGATCCCCAGCTGTTTATGCATAGATAATCTCTCCATTCCCGTGGAACGTTTTTCCTGTTCTTAAGACGTGATTTTGCTGTAGAAGATGGCACTTATAACCAAAGCCCAAAGTGGTATAGAAATGCTGGTTTTTCAGTTTT**CAGGAGTGGGTTGATTTCAG**CACCTACAGTGTACAGTCTTGTATTAAGTTGTTAATAAAAGTACATGTTAAACTTA

**mRNA Transcript:**

CACAGAUGUAAAAGACUUUUUUUUAUACGAUAAAUAACUUUUUUUUAAGUUACACAUUUUUCAGAUAUAAAAGACUGACCAAUAUUGUACAGUUUUUAUUGCUUGUUGGAUUUUUGUCUUGUGUUUCUUUAGUUUUUGUGAAGUUUAAUUGACUUAUUUAUAUAAAUUUUUUUUGUUUCAUAUUGAUGUGUGUCUAGGCAGGACCUGUGGCCAAGUUCUUAGUUGCUGUAUGUCUCGUGGUAGGACUGUAGAAAAGGGAACUGAACAUUCCAGAGCGUGUAGUGAAUCACGUAAAGCUAGAAAUGAUCCCCAGCUGUUUAUGCAUAGAUAAUCUCUCCAUUCCCGUGGAACGUUUUUCCUGUUCUUAAGACGUGAUUUUGCUGUAGAAGAUGGCACUUAUAACCAAAGCCCAAAGUGGUAUAGAAAUGCUGGUUUUUCAGUUUUCAGGAGUGGGUUGAUUUCAGCACCUACAGUGUACAGUCUUGUAUUAAGUUGUUAAUAAAAGUACAUGUUAAACUUA


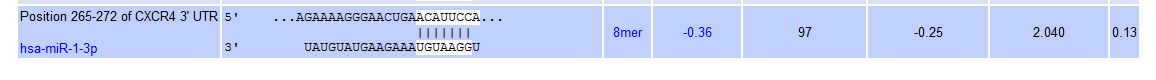


**Primer Sequence:**

**CXCR4 3’UTR Seq (PCR Product: 286)**

**CXCR4F-** 5’-CATATTGATGTGTGTCTAGGC-3’

**CXCR4R-** 5’-CTGAAATCAACCCACTCCTG-3’

**CXCR4 3’UTR Wild Seq (PCR Product: 227)**

**CXCR4WF-** 5’-GGGTCTAGAGGGAACTGAACATTCCAGAG-3’

**CXCR4WR-** 5’-GCCTCTAGACTGAAATCAACCCACTCCTG-3’

**CXCR4 3’UTR Mutant Seq (PCR Product: 227)**

**CXCR4MF-** 5’-GGGTCTAGAGGGAACTGAA**GTAAG**CAGAG-3’

**CXCR4MR-** 5’-GCCTCTAGACTGAAATCAACCCACTCCTG-3’

**Flow cytometry**

1. ***Annexin-V/propidium iodide apoptosis analysis***

SBC5 (-DOX-off), SBC5-DOX-On-miR-1 and NCI-H69(-DOX-off), NCI-H69-DOX-On-miR-1 cells were grown in the presence and absence of DOX for 24-72 h, and annexin-V/PI assay was performed as described previously [8]. Briefly, cells were trypsinized and washed twice with PBS and incubated with Cy5-Annexin-V (Cat# 559934, B.D., Biosciences), and propidium iodide according to the manufacturer’s instructions and stained samples were analyzed by flow cytometry with FlowJo software.

1. ***Cell surface expression of CXCR4***

SBC3, SBC3-miR-1Zip, SBC5, and NCI-H69 cells were grown in RPMI media, whereas SBC5 (-DOX-off), SBC5-DOX-On-miR-1 and NCI-H69(-DOX-off), NCI-H69-DOX-On-miR-1 cells were grown in the presence and absence of DOX for 48 h. Approximately, 1 X 10^5^ cells were collected from each cell line, washed twice with PBS and fixed for 15-20 min using 4% paraformaldehyde. The cells were washed twice with PBS followed by a single wash with antibody binding/FACS buffer (2.0% BSA, 0.1% sodium azide in PBS,). The cells were resuspended in FACS buffer and stained with APC/Cy7 anti-human CD184 (CXCR4) antibodies (Cat# 306528, BioLegend, San Diego, CA, USA) for 1 h at room temperature. Cells were then washed with FACS buffer and finally resuspended in 400µl of FACS buffer for further flow cytometric analysis to determine the cell surface expression of CXCR4. FlowJo was used to analyze the FACS data.

**Immunoblot analysis**

Approximately, 1 X 10^6^ cells were collected by centrifugation at 400 x g, washed with ice-cold PBS, and processed for protein extraction using cell lysis buffer (50 mM Tris-HCl pH 7.5, 250 mM NaCl, 1% NP-40, 0.5% sodium deoxycholate, 0.1% sodium dodecyl sulfate) containing protease and phosphatase inhibitors (Roche, Cat #: 11836153001; Sigma, PhosSTOP Cat#: 4906837001). Whole-cell protein extracts were quantified using a Bio-Rad D/C protein estimation kit. The proteins samples were boiled in Laemmli’s sample buffer, and 40μg of protein (per sample) was resolved by electrophoresis using 8-12% SDS-PAGE. The resolved proteins were transferred to polyvinylidene difluoride membrane, blocked in 5% non-fat milk in PBS with 0.1% Tween-20 (PBS-T) for 1 h, and incubated with the respective primary antibodies **(Supplementary Table…)** overnight at 4°C. Next day, the membranes were washed three times in PBS-T and further probed with appropriate horseradish peroxidase-conjugated secondary antibodies (Invitrogen, diluted at 1:3000-1:4000 in PBS-T with 3% non-fat milk) for 1 h at room temperature, and washed four times in PBS-T. The blots were processed, and bound antibodies were detected using enhanced chemiluminescence detection reagent (Thermo Fisher Scientific, Cat #: 1859698) [9]. The signal was captured onto the X-ray films (Biomax Films, Kodak, NY, USA) or iBright imaging system (Thermo Fisher Scientific, Waltham, MA, USA).

**Immunohistochemistry**

Tissue sections from the resected organs (lung, liver, and brain) of NSG mice bearing metastatic tumors or xenografts from SCLC, and SCLC tissue microarrays (US Bio Lab, Rockville, MD) were baked overnight at 58 ⁰C, deparaffinized in xylene, and rehydrated using higher to lower concentration gradient of alcohol (5 min each). To quench the endogenous peroxide activity, the tissue slides were incubated with hydrogen peroxide in methanol (3% solution), washed in running water for 5-10 min, and antigen retrieval was performed through heating the tissues samples in boiling 0.05 M citrate buffer (pH 6.0) with 0.5% PBST for 10-15 mins. Blocking was performed with 2.5% horse serum (ImmPRESS Universal antibody kit; Vector Laboratories, Burlingame, CA) for 1-2 h at room temperature. The tissue samples were incubated with specific primary antibodies overnight at 4⁰C **(Supplementary Table S6).** Following overnight incubation, the tissue slides were washed twice each with PBST and PBS (10 min per wash) and further incubated for 45 min with anti-mouse/rabbit HRP-conjugated secondary antibodies (Vector Laboratories, ImmPRESS Universal antibody kit for IHC). Finally, the slides were washed twice each with PBST and PBS (10 min each) and the chromogen substrate 3, 3’-diaminobenzidine solution (DAB substrate kit; Vector Laboratories) was used to develop the color. The appearance of brown color indicated a positive expression of the target protein, and following the completion of DAB incubation, the slides were counterstained with hematoxylin. The slides were then washed with water, and dehydrated with ethanol gradient followed by xylene wash, and mounted using Permount (Vector Laboratories, Burlingame, CA). The images were captured at Leica Digital Microscope (DMC4500).

**Table S1. Synthetic oligonucleotides used in this study (miRNAs and DNA probes)**

| **Oligonucleotides** | **Sequence (5’---to---3’)** | **Accession No.^a^** | | **MW (g/mol)** | **T_m_ (°C)** |
| --- | --- | --- | --- | --- | --- |
| miR-1-3P | UGGAAUGUAAAGAAGUAUGUAU | | MI0000437 | 7115.3 | 45.6 |
| Probe-miR-1-3P | **/56-FAM**-ATACATACTTCTTTACATTCCATTT -/**ThioMC3-D/** | | - | 8297.7 | 48.6 |

**^a^**The miRNA accession database No. (<http://www.mirBase.org>).

Abbreviations: [56-FAM: 5’ 6-Carboxyfluorescein. ThioMC3-D/: 3’ Thio-modifier. MW: Molecular weight; T_m_: Melting temperature]. The synthetic miR-1 oligos and DNA-probes specific to the miR-1 are mentioned.

**Table S2. Summary of spectroscopic characterization of AuNPs (UV-Vis, DLS, NTA, TEM)**

| **AuNPs type*^a^*** | **λ_max_^b^**  **(nm)** | **HD (**ø**)*^c^***  **(DLS, nm)** | **PDI*^d^***  **(DLS)** | **Size*^e^***  **(NTA, nm)** | **Size*^f^***  **(TEM, nm)** | **ζ-potential*^g^***  **(mV)** |
| --- | --- | --- | --- | --- | --- | --- |
| 35 nm AuNPs | 534 | 45.8 ± 12 | 0.10 | 39.1 ± 9 | 34.0 ± 2.5 | − 37.2 ± 12 |
| PEG-AuNPs | 534 | 49.3 ± 13 | 0.13 | 47.3 ± 12 | 34.3 ± 2.3 | − 34.1 ± 14 |
| Linker-AuNPs | 534 | 51.3 ± 15 | 0.15 | 48.1 ± 15 | 35.5 ± 2.4 | − 34.5 ± 12 |
| DNA-AuNPs***^h^*** | 535 | 61.2 ± 17 | 0.19 | 61.7 ± 11 | 36.0 ± 2.6 | − 35.1 ± 14 |
| DNA-AuNPs*^i^* | 536 | 63.1 ± 14 | 0.17 | 60.4 ± 17 | 35.4 ± 2.6 | − 34.4 ± 15 |

**Serum-2**

***^a^***Bare and functional AuNPs, ***^b^***Wavelength of maximum absorbance (λ_max_), ***^c^***hydrodynamic diameter (HD, ø). ***^d^***polydispersity index (PDI) that describes the degree of non-uniformity of NPs size distribution. ***^e^***AuNPs size measured using Nanoparticle tracking analysis (NTA). ***^f^***Averaged AuNPs size measured by processing TEM images with Image J. ^g^ζ-potential (surface charge) of AuNPs calculated with Zeta sizer. Results are shown for DNA-AuNPs specific to ***^h^***miR-1-3p; and ***^i^***miR-1-5p. The HD, particle size, and ζ-potential data are represented as means ± SEM in triplicates.

**Table S3. Quantification of the attached DNA-probe per gold nanoparticle (AuNP)**

| **Target miRNA** | **Mole fraction (χ)^a^**  **of PEG-NH_2_** | **[DNA-probe]^b^** | **[AuNP]^c^** | **DNA/AuNP^d^**  **(N_Oligos_)** |
| --- | --- | --- | --- | --- |
| miR-1 | 0.15 | 543 ± 34 | 1.3 | 119 ± 11 |

**^a^**Number of mole fraction of amine-derivatives of thiol-PEG used in passivation. **^b^**Concentration of DNA-probe (nM). **^c^**Concentration of AuNPs (nM). **^d^**Number of attached DNA oligos per nanoparticle (N_Oligos_) calculated by dividing [DNA-probe] by [AuNP].

**Table S4. Summary of proposed optimal conditions tested in miRNA assay**

| **Assay parameters^a^** | **Range of parameters**  **analyzed for assay**  **standardization** | **Optimal**  **parameters** |
| --- | --- | --- |
| Mg^2+^ Conc. (mM) | 0-30 | 5 |
| DSN Conc. (U/µL) | 0.02-0.08 | 0.03 |
| DNA-AuNPs Conc. (nM) | 0-12 | 10 |
| Reaction Time (min) | 0-300 | 15-60 |
| Temperature (⁰C) | 16-60 | 37 |
| Reaction pH | 2-12 | 8 |
| RNAse inhibitor Conc. (U/µL) | 0.02-0.8 | 0.05 |
| Gain (PMT sensitivity) | 80-120 | 120 |

**^a^**List of assay components, parameters and incubation conditions assessed in miRNA detection.

**Table S5.** **Details of antibodies and reagents used in the study.**

| **KEY RESOURCES** | | |
| --- | --- | --- |
| **REAGENT** | **SOURCE** | **IDENTIFIER** |
| **Antibodies** | | |
| FOXM1, WB- 1:1000, IHC-1:400, Chromatin IP-1:100 | Cell Signaling Technology | Cat#20459 |
| RRM2, WB-1:1000, IHC- 1:200 | Cell Signaling Technology | Cat#65939 |
| ERK1/2, WB-1:1000 | Cell Signaling Technology | Cat#9102 |
| p-ERK (Thr-202/Tyr-204), WB:1:1000 | Cell Signaling Technology | Cat#4370 |
| Akt, WB:1:1000 | Cell Signaling Technology | Cat#2920 |
| p-Akt (Ser-473), WB:1:1000 | Cell Signaling Technology | Cat#4060 |
| CXCR4, IHC-1:300 | Abcam | Cat#ab124824 |
| APC/Cyanine7 anti-human CD184 (CXCR4), for flow cytometry | BioLegend | Cat#306528 |
| Ki67, IHC-1:100 | Abcam | Cat#ab92742 |
| CD31, WB-1:1000 | Cell Signaling Technology | Cat#77699 |
| β-actin, WB-1:10,000 | Sigma | Cat#A1978 |
| Snail, WB-1:1000 | Cell Signaling Technology | Cat #3879 |
| Zeb-1, WB-1:1000 | Cell Signaling Technology | Cat#70512 |
| **Kits and chemicals** | | |
| miRNeasy serum/plasm kit | Qiagen | Cat # 217184 |
| Total RNA isolation kit mirVana™ | Invitrogen™, Thermo Scientific, USA | Cat#AM1561 |
| MagMAX™ mirVana™ | Invitrogen™, Thermo Scientific, USA | Cat#A27828 |
| Stem-loop primers specific for miR-1-3p | Applied Biosystem | Assay ID: 4427975 |
| RNU6B | Applied Biosystem | Assay ID: 4427975 |
| TaqMan Universal PCR Master Mix | Applied Biosystem | Cat#4304437 |
| BaseScope^TM^ Detection Reagent V2 | Advanced Cell Diagnostics | Cat#323910 |
| BaseScope^TM^ Probe BA-Hs-pre-MIR-1-1zz-st | Advanced Cell Diagnostics | Cat#723671 |
| BaseScope^TM^ Probe BA-DapB-1zz | Advanced Cell Diagnostics | Cat#701021 |
| BaseScope^TM^ Probe BA-Hs-PPIB-1zz | Advanced Cell Diagnostics | Cat#710171 |
| ImmPRESS Universal Polymer kit, Peroxidase | Vector Laboratories | Cat#MP-7500 |
| DAB Peroxidase (HRP) Substrate Kit (with Nickel), 3,3’-diaminobenzidine | Vector Laboratories | Cat#SK-4100 |
| FDI-6 | Millipore Sigma | Cat#533259 |
| AMD3100 | Selleck Chemical | Cat#S8030 |

**References**

1. Maus, L., J.P. Spatz, and R. Fiammengo, *Quantification and reactivity of functional groups in the ligand shell of PEGylated gold nanoparticles via a fluorescence-based assay.* Langmuir, 2009. **25**(14): p. 7910-7.

2. Degliangeli, F., et al., *Absolute and direct microRNA quantification using DNA-gold nanoparticle probes.* J Am Chem Soc, 2014. **136**(6): p. 2264-7.

3. Kshirsagar, P., et al., *DNA-gold nanoprobe-based integrated biosensing technology for non-invasive liquid biopsy of serum miRNA: A new frontier in prostate cancer diagnosis.* Nanomedicine, 2022. **43**: p. 102566.

4. Siddiqui, J.A., et al., *GDF15 promotes prostate cancer bone metastasis and colonization through osteoblastic CCL2 and RANKL activation.* Bone Res, 2022. **10**(1): p. 6.

5. Mirzapoiazova, T., et al., *Protein Phosphatase 2A as a Therapeutic Target in Small Cell Lung Cancer.* Mol Cancer Ther, 2021. **20**(10): p. 1820-1835.

6. Chaudhary, S., et al., *Overexpression of caspase 7 is ERalpha dependent to affect proliferation and cell growth in breast cancer cells by targeting p21(Cip).* Oncogenesis, 2016. **5**: p. e219.

7. Kanchan, R.K., et al., *MiR-1253 exerts tumor-suppressive effects in medulloblastoma via inhibition of CDK6 and CD276 (B7-H3).* Brain Pathol, 2020. **30**(4): p. 732-745.

8. Khan, P., et al., *Elucidation of Dietary Polyphenolics as Potential Inhibitor of Microtubule Affinity Regulating Kinase 4: In silico and In vitro Studies.* Sci Rep, 2017. **7**(1): p. 9470.

9. Khan, P., et al., *Luminol-based chemiluminescent signals: clinical and non-clinical application and future uses.* Appl Biochem Biotechnol, 2014. **173**(2): p. 333-55.
